# Benzylsuccinate Synthase is Post-Transcriptionally Regulated in the Toluene-Degrading Denitrifier *Magnetospirillum* sp. Strain 15-1

**DOI:** 10.3390/microorganisms8050681

**Published:** 2020-05-07

**Authors:** Ingrid Meyer-Cifuentes, Sylvie Gruhl, Sven-Bastiaan Haange, Vanessa Lünsmann, Nico Jehmlich, Martin von Bergen, Hermann J. Heipieper, Jochen A. Müller

**Affiliations:** 1 Department of Environmental Biotechnology, Helmholtz Centre for Environmental Research - UFZ, Permoserstr. 15, 04318 Leipzig, Germany; ingrid.meyer.cifuentes@dsmz.de (I.M.-C.); sylvie.gruhl@outlook.de (S.G.); jochen.mueller@ufz.de (J.A.M.); 2 Junior Research Group of Microbial Biotechnology, Leibniz Institute DSMZ, German Collection of Microorganisms and Cell Cultures, Inhoffenstr. 7B, 38124 Braunschweig, Germany; 3 Department of Molecular Systems Biology Helmholtz Centre for Environmental Research-UFZ, Permoserstr. 15, 04318 Leipzig, Germany; sven.haange@ufz.de (S.-B.H.); vanessa.luensmann@posteo.de (V.L.); nico.jehmlich@ufz.de (N.J.); martin.vonbergen@ufz.de (M.v.B.); 4 Group of Functional Proteomics, Institute of Biochemistry, Faculty of Biosciences, Pharmacy and Psychology University of Leipzig, Talstrastr. 33, 04103 Leipzig, Germany

**Keywords:** toluene, nitrate, benzylsuccinate synthase, *Magnetospirillum*, rhizosphere

## Abstract

The facultative denitrifying alphaproteobacterium *Magnetospirillum* sp. strain 15-1 had been isolated from the hypoxic rhizosphere of a constructed wetland model fed with toluene. This bacterium can catabolize toluene anaerobically but not aerobically. Here, we used strain 15-1 to investigate regulation of expression of the highly oxygen-sensitive glycyl radical enzyme benzylsuccinate synthase, which catalyzes the first step in anaerobic toluene degradation. In cells growing aerobically with benzoate, the addition of toluene resulted in a ~20-fold increased transcription of *bssA*, encoding for the catalytically active subunit of the enzyme. Under anoxic conditions, *bssA* mRNA copy numbers were up to 129-fold higher in cells growing with toluene as compared to cells growing with benzoate. Proteomics showed that abundance of benzylsuccinate synthase increased in cells growing anaerobically with toluene. In contrast, peptides of this enzyme were never detected in oxic conditions. These findings show that synthesis of benzylsuccinate synthase was under stringent post-transcriptional control in the presence of oxygen, which is a novel level of regulation for glycyl radical enzymes.

## 1. Introduction

Microbial toluene degradation has been extensively studied, resulting in the detailed description of one anaerobic and various aerobic catabolic pathways for this aromatic compound [1,2,3,4]. Anaerobic degradation is initiated by benzylsuccinate synthase (BSS, encoded by *bss* genes), which adds a molecule of fumarate to the methyl group of toluene [5,6]. The generated benzylsuccinate is transformed to benzoyl-CoA in a β-oxidation-like scheme involving enzymes encoded by the *bbs* genes [7]. Benzoyl-CoA is then reductively dearomatized either by an ATP-dependent Class I benzoyl-CoA reductase (BcrABCD) in facultative anaerobes or by a non-homologous ATP-independent Class II reductase in various strict anaerobes [8,9]. Further transformation yields central metabolites. 

BSS is an (αβγ)_2_ heterohexamer and belongs to the family of glycyl radical enzymes (GREs), class II [5,10,11,12]. The large α subunit (BssA) when activated contains the glycyl radical [6], whereas the small β (BssB) and γ (BssC) subunits are potentially needed for formation, solubility and stability of a catalytically competent enzyme [5,6]. The glycyl radical is generated post-translationally by an activating enzyme, BssD [5,6]. Glycyl radical chemistry is incompatible with oxic conditions. The active form of BSS is oxygenolytically cleaved at the glycyl radical site [13]. Its inactive form is already oxygen sensitive, likely due to the presence of a [4Fe4S]-cluster of apparently very low midpoint potential in each of the small subunits [14,15]. 

The genomic organization of *bss* and *bbs* genes has been described in ample detail [1,8,16,17]. Common to all sequenced anaerobic toluene degraders are *bssDCABEF* and *bbsABCDEFGH* gene clusters with shared syntenies. The genes *bssE* and *bssF* encode for members of the AAA+ and von Willebrand factor protein superfamilies, respectively, but their precise function is uncertain. All sequenced denitrifying and some sulfate-reducing toluene degraders harbour also the *bssGIJKL* genes which have only general function predictions, and several strains carry additional genes apparently associated with anaerobic toluene transformation such as *bssH*, predicted to encode for a transporter, and various *xylR*-type regulators.

There is some genetic knowledge on regulation of expression of the *bss* and *bbs* genes, but sensory inputs are not well defined. In denitrifying strains, expression of apparently both gene clusters is controlled by a two-component regulatory system encoded by the nearby located *tdiSR* genes [18,19,20]. The physiologically important inducer for this system could be either toluene or benzylsuccinate [16,21,22]. The regulatory impact of oxygen is uncertain and may even differ among strains [16,23,24]. It was furthermore proposed that in denitrifiers the level of gene expression is linked to the availability of the terminal electron acceptor nitrate [25]. An improved understanding of regulatory schemes will help to better predict anaerobic toluene degradation in the environment. 

We have been investigating microbial in situ degradation of toluene in the rhizosphere of Planted Fixed-bed Reactors (PFRs) [26], which are model systems for analysing processes taking place in constructed wetlands [27]. Although the pore water of the PFRs was typically oxic and protein-based stable isotope probing provided clear evidence for aerobic but not for anaerobic toluene metabolism, *bssA* transcript levels surpassed the abundance of every quantified transcript required for aerobic toluene degradation by at least an order of magnitude [27]. Metagenomics and sequencing of *bssA* clone libraries indicated that almost all of those transcripts were generated by members of the genus *Magnetospirillum*, which constituted up to 6% of the rhizospheric bacterial community. Yet there was no metaproteomics evidence for the presence of BSS in the PFRs even so various peptides of likely *Magnetospirillum* origin, including some derived from benzoyl-CoA reductase, were found [27]. Subsequently, a representative anaerobic toluene degrader, *Magnetospirillum* sp. 15-1, was isolated from a PFR and a draft genome sequence of this microbe was generated [28,29]. A physiological characterization, together with an extensive genomic analysis, revealed that the strain degrades toluene anaerobically but not aerobically.

The key aim of this study was to investigate the effect of oxygen and toluene on *bss* gene expression and synthesis of BSS in growing cells of *Magnetospirillum* sp. 15-1. Furthermore, the expression of BSS was studied under nitrate-reducing conditions with various nitrate concentrations.

## 2. Materials and Methods 

### 2.1. Growth Medium and Cultivation

All cultivations of strain *Magnetospirillum* sp. 15-1 were performed in a mineral medium previously described by Tschech and Fuchs [30]. For anaerobic cultivation of strain 15-1, the medium was prepared anoxically with either toluene or benzoate as the sole carbon source and various concentrations of nitrate as the electron acceptor. The medium was amended with the reducing agent Na_2_S (0.25 mM) and the redox indicator resazurin (0.8 µM). Subsequently, 100 mL each of the mineral medium was filled into 240 mL-serum bottles and flushed with N_2_ for 20 min. The bottles were then sealed with teflon-coated stoppers, crimped and autoclaved. FeSO_4_, MgCl_2_, CaCl_2_, and vitamins were then added from anoxic and sterile stock solutions. For aerobic cultivation, benzoate (2.5 mM) was always used as the sole carbon and electron source since strain 15-1 is unable to use toluene under oxic conditions [29]. Under this condition, Na_2_S, resazurin and KNO_3_ were omitted, cultures were shaken and aseptic gaseous exchange with the ambient atmosphere was permitted. Aeration for aerobic cultivation was assured by using only 50 mL of the mineral media in 240 mL serum bottles. For anaerobic and aerobic cultivation, the pH was adjusted to 7.1 with 1M NaOH. Incubations were performed in triplicate or duplicate at 30 °C and bacterial growth was assessed by measuring optical density with a spectrophotometer (UV/VIS Spectrometer Lambda 2S, Perkin-Elmer, Waltham, MA, USA) at 560 nm.

To record gene expression and proteins synthesis under oxic conditions, cultures were supplied with benzoate (2.5 mM) as the carbon source to promote bacterial growth. In the following the abbreviation AB is used for this cultivation condition. Toluene alone (0.3 mM) (AB_T) and together with 5 mM KNO_3_ (AB_TN) were tested as inducers. Benzoate alone (2.5 mM) was used as the reference (AB_B) culture. Cultures growing exponentially with 5 mM benzoate as sole carbon source under oxic conditions were used as pre-inoculum. Under anoxic condition various concentrations of nitrate (2.5–10 mM KNO_3_) were tested with 0.5 mM toluene. Cells grown with 2.5 mM benzoate supplied with 2.5–10 mM KNO_3_ were used as the reference. In the following the abbreviation AN is used for this cultivation condition. For the various nitrate concentrations and 0.5 mM toluene, the cultures were designated as: AN_T2.5 (2.5 mM KNO_3_); AN_T5 (5 mM KNO_3_) and AN_T10 (10 mM KNO_3_). These cultures were incubated in parallel with their corresponding reference culture containing the various nitrate conditions and benzoate alone: AN_B2.5, AN_B5 and AN_B10. Cultures growing exponentially with 1 mM toluene and 10 mM KNO_3_ under anoxic conditions were used as pre-inoculum. For the pre-inoculum, toluene and nitrate were supplied before depletion. Thereby, cultures received twice 0.5 mM toluene and 5 mM KNO_3_. The first injection was supplied after bacterial inoculation in fresh media and the second injection after depletion of toluene. Injections of toluene were performed with a 10 µL Hamilton syringe 801 RN (Hamilton, Reno, NV, USA). Depletions of toluene and nitrate were measured as described before [29]. 

AN cultures were harvested in exponential phase before toluene and nitrate depletion. Harvesting points were chosen based on growth experiments in which toluene, benzoate, nitrate, and nitrite were measured [29] (Section S2.1). Bacterial growth and organic carbon consumption are shown in Figure S1. 

### 2.2. RNA Extraction and cDNA Synthesis

For RNA isolation, cells of strain 15-1 were harvested at late exponential phase (OD_560_ 0.13 to 0.16) by centrifugation for 15 min at 8000 rpm and room temperature. RNA extraction was performed by using the RNeasy Mini Kit (Qiagen, Hilden, NW, Germany) according to the manufacturer´s instructions with some modifications. Cell lysis (after resuspension in RLT) was carried out by transferring the samples into FastPrep^®^ tubes containing lysing beads-Matrix B (MP Biomedicals, Santa Ana, CA, USA) and disrupting them in a Fastprep instrument (FastPrep^®^-24 *Classic*, MP Biomedicals, Santa Ana, CA, USA) for 40 s at 6 m/s. To remove possible DNA contamination, the RNA samples were treated twice with DNase I (RNase-Free Dnase kit, Qiagen, Hilden, NW, Germany) and then purified with the RNA Cleanup protocol (RNeasy Mini Kit, Qiagen, Hilden, NW, Germany) following manufacturer’s instructions. RNA concentration was measured by using Quant-iT™ RiboGreen^®^ RNA Reagent and Kit (Invitrogen, Carlsbad, CA, USA) according to the manufacturer´s instructions. To assess the quality of isolated RNA, the samples were loaded in an 1.2% agarose gel and measured in a Nanodrop (Nanodrop ND-1000 Spectrophotometer, Thermo Fisher Scientific, Waltham, MA, USA). The purified RNA samples were free of salt and phenol contamination. DNA contamination was additionally investigated by performing 16S rRNA amplification from the RNA elutes (Section S2.2) and loaded in an 1.2% agarose gel. 

The purified RNA (150 ng) was subjected to cDNA synthesis by using an Ominiscript^®^ Reverse Transcription Kit (Qiagen, Hilden, NW, Germany) with random hexamer primers according to manufacturer’s instructions. A negative control without reverse transcriptase was prepared per sample. All samples were stored at −20 °C until usage (Section S2.3).

### 2.3. Transcriptional Organization of bss, tdi, and bbs Gene Clusters by Reverse-Transcriptase PCR (RT-PCR) 

cDNAs of the intergenic regions of *bss*, *tdi*, and *bbs* genes as well as a fragment of the *xylR* transcript were prepared (Section S2.3) and PCR-amplified with the primers listed in Table 1. The amplification mixtures contained l µL of cDNA, 6.25 µL Red Taq 2 × MasterMix (VWR, Darmstadt, HE, Germany), 0.5 µL of each primer (10 µM stock concentration), and 4.25 µL of ddH_2_O. Amplification conditions were as follows: 2 min at 95 °C, followed by 30 cycles of annealing for 30 sec at 60 °C, elongation for 30 sec at 72 °C and denaturation for 30 sec at 96 °C. Final elongation was done at 75 °C for 5 min. 

### 2.4. Gene Expression Assessment by Quantitative RT- PCR (qRT-PCR)

The quantification of *bssA* and *bcrC* gene expression in anoxic and oxic conditions was carried out by using the primer pairs bssA_F3/bssA_R3 and 2bcrC_F/2bcrC_R. These primer pairs annealed to intergenic sections of the *bssA* and *bcrC* genes, respectively (Table 1). Amplification conditions were the following: 2 min at 95 °C, 40 cycles of annealing for 20 sec at 58 °C (*bssA*), 62 °C (*bcrC*) and 56 °C (16s rRNA). Elongation was carried out for 20 sec at 72 °C and denaturation for 3 sec at 95 °C. Final elongation was done at 75 °C for 5 min. For the normalization of *bssA* and *bcrC* transcripts in each sample, 16S rRNA copy numbers were recorded after qRT-PCR by using the primer pair 16S_F/16S_R. The quantification was performed in a 96-well Microtiter microplate on a Step One Plus Real Time PCR System (Applied Biosystems, Thermo Fischer Scientific, Waltham, MA, USA) using the KAPA SYBR Fast master mix (Section S2.5). In the wells, standards and cDNA samples were prepared and measured in triplicate. A negative control (template without RT) per sample was also included. The standards were serially diluted, and the cDNA samples were either undiluted or diluted 1:10 depending on the experimental conditions (Section S2.5). 

Quantification was performed by calculating the abundance of *bssA* and *bcrC* transcripts from their corresponding standard curves (Section S2.4). Normalization was carried out by using 16S rRNA copy numbers as the calibrator. Fold changes were further calculated by using the expression of *bssA* and *bcrC* from benzoate-grown cells as the reference (Section S2.6).

### 2.5. Protein Extraction and Preparation

For protein extraction, 25 mL of culture was harvested in late exponential phase (OD_560_ 0.13 to 0.16) and centrifuged for 10 min at 8000 rpm. The pellets were then resuspended in 500 µL of Urea buffer (8 M urea and 2 M thiourea) and transferred to FastPrep tubes containing 0.1 mm zirconia and 2.85–3.45 mm glass beads (Carl Roth, Karlsruhe, BW, Germany). The cells were lysed in a FastPrep device for 40 s at 5 m/s. (FastPrep^®^-24 *Classic*, MP Biomedicals, Santa Ana, CA, USA) and by sonication on ice twice at 70 % power (25 W) and 70 % duty cycle (UP50H, Hielscher Ultrasonic, Teltow, BB, Germany).

The total amount of protein was quantified by the Bradford method as described before [33]. Then, 60 µg of proteins from the supernatants were precipitated with a 5-fold volume of acetone (100%) and incubated overnight a −20 °C. After incubation, the protein pellets were precipitated by centrifugation (14,000 rpm for 10 min) and air-dried in a vacuum concentrator (Eppendorf, Hamburg, HH, Germany) for 5 min. Protein separation, staining and proteolytic cleavage was performed as described before [34]. Peptide lysate desalting was performed using SOLAµ HRP 96-well plates (Thermo Fischer Scientific, Waltham, MA, USA) by applying different concentrations of acetonitrile and formic acid. Prior to LC-MS/MS measurement, proteotypic peptide samples were quantified via NanoDrop (NanoDrop 2000C, Thermo Fischer Scientific, Waltham, MA, USA).

### 2.6. LC-MS/MS and Proteome Analysis

The samples were injected into a Nano-HPLC (Ultimate nanoRSLC 3000, Thermo Fischer Scientific, Waltham, MA, USA) and proteotypic peptides lysates were separated with a C18-reverse phase analytical column (Acclaim PepMap^®^ 100, 75 µm × 25 cm, particle size 3 µM, nanoViper, Thermo Fisher Scientific, Waltham, MA, USA) in a two-step LC gradient. The eluting peptide lysates were ionized by a nano-ion source (TriVersa Nanomate, Advion, Ithaca, NY, USA) and measured using a Q Exactive HF mass spectrometer (Thermo Fisher Scientific, Waltham, MA, USA) with the setup described in the Supplemental Material Section S2.7.

The raw LC-MS/MS data were analysed using Proteome Discoverer v. 2.2 (Thermo Fischer Scientific, Waltham, MA, USA) with the following parameters: oxidation for dynamic modifications, carbamidomethylation for static modifications, MS tolerance 10 ppm, MS/MS tolerance 0.02 Da, trypsin (set as full specific) and two missed cleavage sides. Database search was performed using Sequest HT algorithm against the annotated protein-coding sequence database of *Magnetospirillum* sp. 15-1 [28] from RAST [35,36,37]. Only peptides with a false discovery rate (FDR) < 1% calculated by Percolator [38] were considered as identified. Identified proteins were grouped by applying the strict parsimony principle, in which protein hits are reported as the minimum set that accounts for all observable peptides. Protein abundances were then calculated based on the top3 approach implemented in Proteome Discoverer v. 2.2 and normalization of the peptides were manually performed as described before [39,40]. Proteome fold changes of toluene-grown cells were calculated relative to benzoate-grown cells, for aerobic and anaerobic conditions separately as shown in Table S3 and as described in the Section S2.8. Venn diagrams were generated using the Venn Diagram Plotter [41]. Functional proteome analysis was performed using GHOSTKOALA [42] and IPath [43,44]. MeV [45] was used for hierarchical clustering.

### 2.7. Data Availability

The qPCR and proteomic raw data have been deposited in Figshare [46]. Data sets S1 [47], S2 [48] and S3 [49] are included. 

## 3. Results

### 3.1. Genomic Organization of Genes Involved in Anaerobic Toluene Transformation

**Figure 1:**
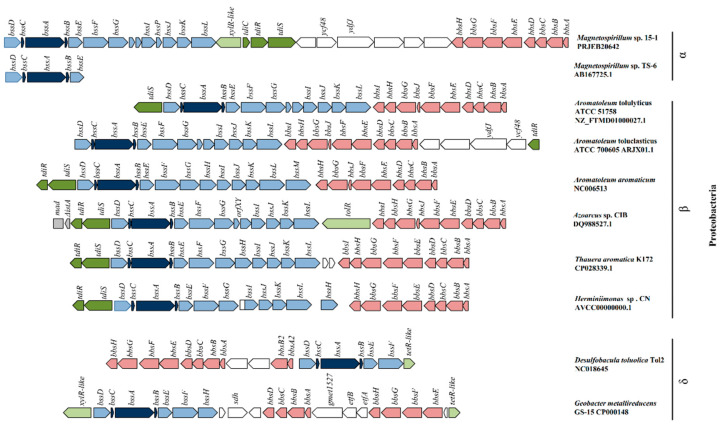
Organization and comparison of the *bss–bbs* gene region in selected bacteria capable of anaerobic toluene degradation. Genes are represented by arrows: ‘dark blue’ for *bssABC*; ‘light blue’ for accessory *bss* genes; ‘pink’ for genes needed for transformation of benzylsuccinate to benzoyl-CoA; ‘olive’ for *tdi* genes; ‘light green’ for putative regulator-coding genes; ‘white’ for remaining genes without evidence for involvement in anaerobic toluene degradation. The gene names are as follows: *bssABC* and *bssD* subunits code for a benzylsuccinate synthase and its corresponding activase, respectively; *bssE*, putative chaperone; *bssG-J* and *bssL*, unknown function; *bssK*, mRNA binding protein; *xylR*, σ^54^-dependent regulator, *tdiC*, regulator of TdiR activity; *tdiR*, toluene degradation regulator; *tdiS*, toluene degradation inducing sensor; *bbsEF*, (3-methyl) benzylsuccinate CoA transferase; *bbsG*, (3-methyl) benzylsuccinyl-CoA dehydrogenase; *bbsH*, (3-methyl) phenylitaconyl-CoA hydratase; *bbsCD*, 2-[hydroxyphenyl(methyl)]-succinyl-CoA dehydrogenase; *bbsAB*, BbsAB, (3-methyl) benzoylsuccinyl-CoA thiolase.

It was previously hypothesized that horizontal gene transfer events of *bss* genes had occurred among denitrifiers [15,16]. The closely related strains 15-1 and TS-6 [23] are the only known carriers of *bss* and *bbs* genes within the alphaproteobacterial genus *Magnetospirillum*. These two strains group phylogenetically with the non-toluene-degrading *M. magnetotacticum* and *M. magneticum* [23,28,29]. Thus, it is most parsimonious to assume that an ancestor of strains 15-1 and TS-6 acquired horizontally the genes needed for anaerobic toluene degradation. The analysis of sequence composition indicated that the *bss* and *bbs* genes of strain 15-1 share a common ancestor with those of various toluene-degrading *Azoarcus* sp.and *Aromatoleum* spp. isolates and the uncultivated *Herminiimonas sp.* CN [50]. The gene content of the *bss* cluster is similar in strain 15-1, *Azoarcus sp.* CIB, *Aromatoleum tolulyticus* ATCC 51758, and *Aromatoleum toluclasticus* ATCC 700605 except for a gene, *bssP*, which is unique to *Magnetospirillum* sp. 15-1. The *bssP* gene is located between *bssI* and *bssJ* in strain 15-1 and is predicted to encode a small protein of unknown function (Figure 1). Amino acid identities of BssA and BssE ranged from 79 to 86% between strain 15-1 and the denitrifiers mentioned above (Table S1). Identities of the other predicted proteins coded by the *bss* genes ranged from 48 to 76% between strain 15-1 and the respective closest relative. The *bbs* genes needed for the transformation of benzylsuccinate into benzoyl-CoA are present in all denitrifying toluene degraders except for *bbsJ* and *bbsI*, which are of unknown function and are absent in several strains including 15-1. Identities of the Bbs proteins ranged from 73 to 84% between strain 15-1, *Azoarcus sp.* CIB, and *Herminiimonas* sp. CN except for BbsC, which shared at most only 45% amino acid sequence identity between strain 15-1 and the other strains (confirmed by Sanger sequencing on the nucleic acid level). Identities of TdiS and TdiR were around 60% between strain 15-1 and *Herminiimonas* sp. CN, the host of their closest homologs. Among the TdiS homologs most of the sequence divergence was in the second PAS/PAC domain (involved in dimerization) of the protein [16,51] while TdiR divergence was more evenly distributed over the entire sequence length (Figure S2). The GC% of the *bss* cluster (62.8%) is slightly lower than that of the *bbs* cluster (65.2%) and the whole genome (65.6%) of strain 15-1.

There are a few differences between strain 15-1 and all other sequenced denitrifying toluene degraders at this genomic locus. In strain 15-1, the *tdi* genes are located between the *bss* and *bbs* genes rather than upstream of *bssD*, they are in reverse order, and 21 bp upstream of *tdiR* is a gene predicted to encode a protein of 149 amino acid residues with a cystathionine-beta-synthase (CBS) domain. CBS domains may play a regulatory role via making proteins sensitive to adenosyl carrying ligands such as AMP, ATP and *S*-AdoMet [52]. Protein alignment by BLAST revealed that close homologues to the gene in strain 15-1 are present at various genomic loci in all sequenced *Magnetospirillum* strains and other Alphaproteobacteria, in particular in strains isolated from rhizospheres, but not in other Proteobacteria (data not shown). Between the CBS domain-encoding gene and the *bss* cluster is a gene predicted to encode a σ^54^-dependent NtrC/XylR-type transcriptional regulator with around 42% amino acid sequence identity with a protein coded adjacent to the *bss* gene locus in toluene-degrading *Geobactereaceae*. The various genetic arrangements show on one side congruity among denitrifying toluene degraders concerning basic features and some regulatory aspects of benzylsuccinate generation and its transformation into benzoyl-CoA, but indicate also that these mosaic genetic structures result in strain-specific differences in regulation, enzyme assembly, and even catalysis [53].

### 3.2. Regulation of bss Gene Expression in Magnetospirillum sp. Strain 15-1

The transcriptional organization of the *bss*, *tdi* and *bbs* genes was investigated by RT-PCR with RNA isolated from toluene-grown cells (Figure S3, primer sequences provided in Table 1).

RT-PCR products of the expected sizes were obtained for all intergenic regions of the *bss* and *bbs* cluster, demonstrating that these genes form two polycistronic units. Likewise, the gene encoding a CBS domain protein was co-transcribed with *tdiSR*. We name this novel gene *tdiC* and propose that its product is involved in modulating the activity of TdiR.

The previously proposed regulatory DNA sequence (ARGTGTYCGCACC) [16] was found 121 bp and 109 bp upstream of the predicted starts of *bssD* and *bbsA*, respectively, suggesting that the *bss* and *bbs* genes are co-regulated in strain 15-1. To identify environmental parameters that play a role in the regulation of expression of these genes in strain 15-1, we calculated fold changes after RT-qPCR quantification from total RNA isolated from cultures grown under aerobic and anaerobic conditions, in the presence of toluene and benzoate, and various concentrations of nitrate (Data Set S1 and S2). The expression of benzoyl-CoA reductase was recorded as reference by measuring the *bcrC* gene. 

**Figure 2:**
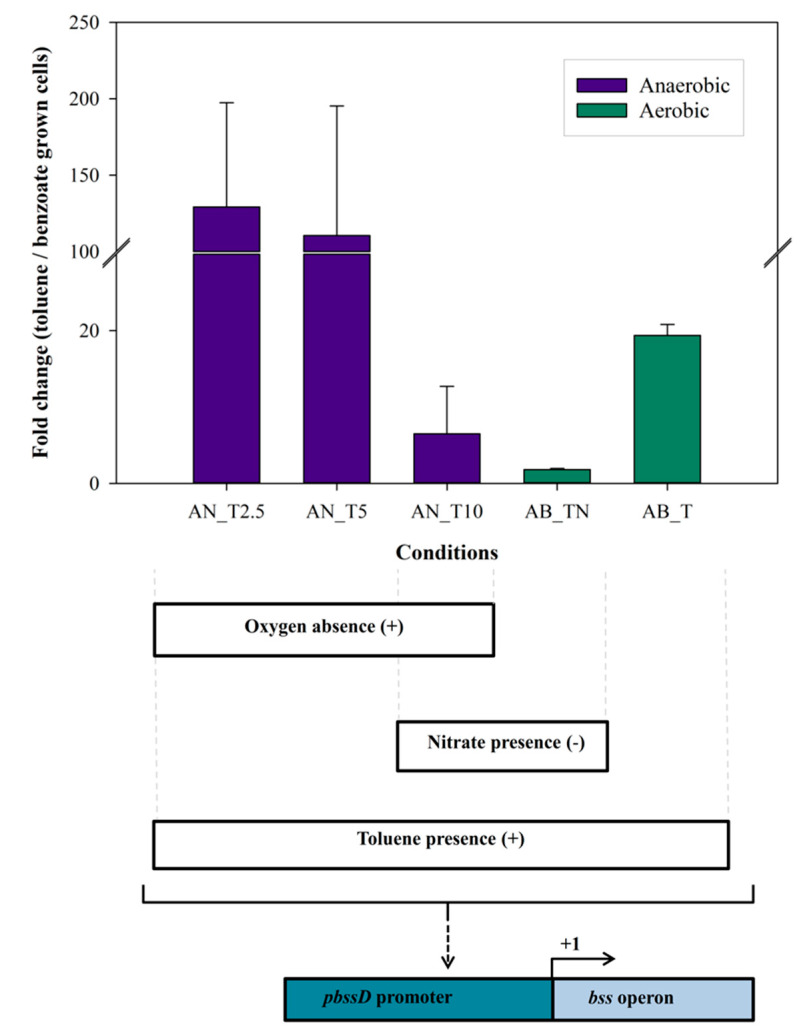
*bssA* expression in anaerobic and aerobic cultures of *Magnetospirillum* sp. 15-1. AN_T represents cultures growing anaerobically with toluene and either 2.5 mM (AN_T2.5), 5 mM (AN_T5), or 10 mM (AN_T10) of KNO_3_. AB_T represents cultures growing aerobically with benzoate and supplied additionally with either toluene alone (AB_T) or toluene and 5 mM of KNO_3_ (AB_TN). The promoter (*pbssD* of the *bss* operon) and the initiation of its transcription (+1) could be induced (**+**) or repressed (–) due to the presence of oxygen, nitrate, and toluene.

In cells growing aerobically on benzoate, the addition of toluene induced *bssA* expression by about 20-fold (Figure 2). This result corresponded to the high *bssA* transcript levels in the oxic PFRs [27,34]. The range of anaerobic *bssA* induction was queried for via differential comparison of cultures grown either with toluene or with benzoate as carbon and electron donor. There was an induction of *bssA* by 119-fold in toluene-grown cells relative to benzoate-grown cells (Figure 2). This further increase in gene expression as compared to the effect of toluene alone was likely triggered by the absence of molecular oxygen. This signal may potentially be sensed directly via an FNR-type scheme (regulator of fumarate and nitrate reduction) as with the well-studied GRE formate C-acetyltransferase (pyruvate formate lyase) [54,55], or indirectly via benzylsuccinate formation as proposed effector for the Tdi regulatory system [16,21], or via the presence of nitrate detected by sensors-regulators such as NarXL and NarQP [56,57,58,59]. 

To further illuminate the question of sensory input we searched for potential regulatory components in the draft genome of strain 15-1. We identified 12 genes coding for members of the CRP/FNR superfamily (Table S2) and at least 10 two-component regulatory systems where the response regulator belongs to the NarL/FixJ family. The canonical binding motifs for FNR [60] and for the sensor-regulator system NarP/NarL [58,61] were not detected in the upstream regions of the *bss* and *bbs* genes. Yet the FNR binding motif was found only 4 times in the genome of strain 15-1, and those hits were not located next to classical genes of the FNR regulon such as *nar* and *nir* genes [62], indicating a deviation from the consensus binding sequence in this strain. Binding motives for NarL/NarP were found upstream of *nar* genes. Thus, there was no genomic evidence for an involvement of nitrate-sensing in *bss* and *bbs* expression, while sequence analyses were inconclusive in regard to a regulatory mechanism encompassing oxygen-sensing by an FNR-like scheme.

To test experimentally whether nitrate induces *bssA* expression, strain 15-1 was cultivated under anoxic conditions with toluene and benzoate at various concentrations of nitrate (2.5, 5, and 10 mM). In addition, we calculated *bssA* fold changes from cultures grown aerobically with benzoate in the co-presence of 5 mM nitrate and 0.3 mM toluene (Figure 2). Under anoxic conditions, the growth rate with 2.5 mM and 5 Mm nitrate were 0.05 h^−1^ and 0.07 h^–1^ respectively, while with 10 mM nitrate the growth rate was reduced to 0.02 h^-1^. Nitrite was never detected in any of those cultures (limit of detection: 21 µM). The relative abundance of *bssA* transcripts in toluene-grown cultures versus benzoate-grown cultures with 2.5 mM nitrate was similar (129-fold induction) to that with 5 mM nitrate (119-fold induction). In contrast, with 10 mM nitrate the *bssA* gene was induced only 7-fold in cells grown anaerobically with toluene. The level of *bcrC* transcription was rather similar at the various nitrate concentrations in both anaerobically- and aerobically-grown cells (Figure S4). In aerobically-grown cultures, the level of *bssA* induction was even lower in the presence of nitrate than without it (Figure 2). Thus, our data support that nitrate-sensing is unlikely to be involved in up-regulation of *bss* expression. 

### 3.3. Proteins Involved in Anaerobic Toluene Degradation 

**Figure 3:**
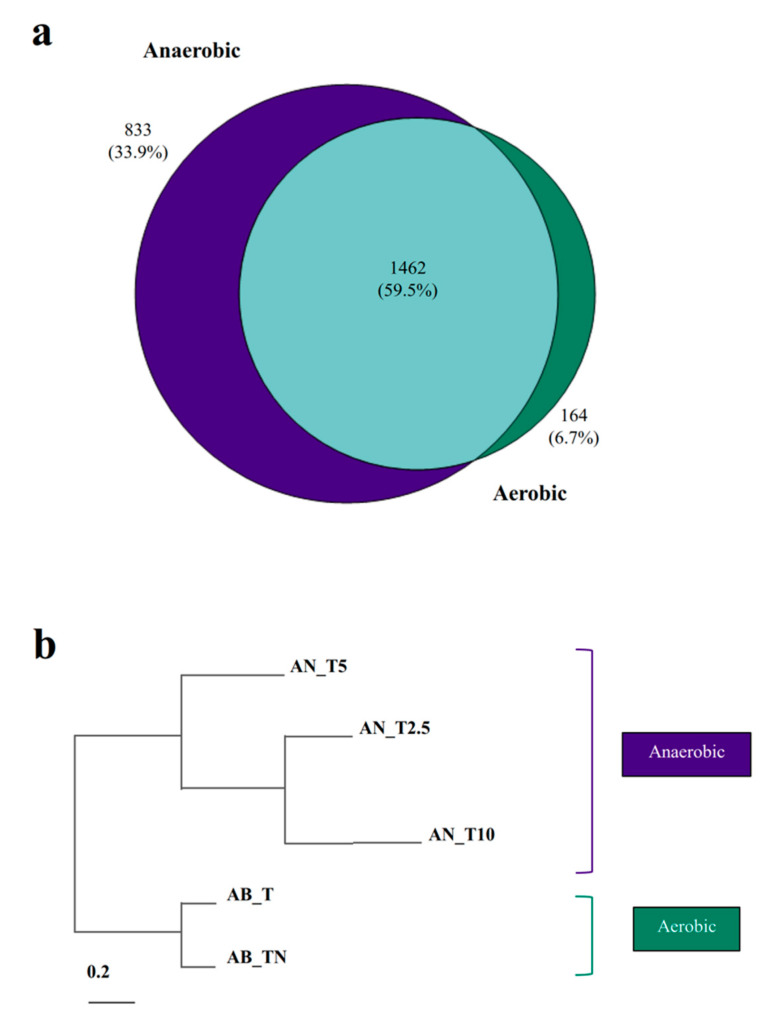
Proteome changes in response to oxygen and nitrate. Total and shared proteins (**a**) and hierarchical clustering of proteome profiles (**b**) among oxic and anoxic conditions with different nitrate concentrations are shown. AN_T represents cultures growing anaerobically with toluene and either 2.5 mM (AN_T2.5), 5 mM (AN_T5), or 10 mM (AN_T10) of KNO_3._ AB_T represents cultures growing aerobically with benzoate and supplied additionally with either toluene alone (AB_T) or toluene and 5 mM of KNO_3_ (AB_TN).

Next, we measured the proteomes based on peptide identification of strain 15-1 grown under the same conditions as in the experiments targeting gene expression. The genome of strain 15-1 harbors 5,095 predicted coding sequences (CDS). In total, 2,459 unique protein groups were identified where 59% were detected under both anoxic and oxic conditions (Figure 3a, Data set S3). The proteome coverage is similar to that of *Escherichia coli* under 22 experimental conditions [63]. A hierarchical clustering dendrogram based on protein log_2_ fold-changes showed the expected topology with branches for the anaerobic and the aerobic cultures (Figure 3b). KEGG pathway mapping revealed only few differences between anoxic and oxic conditions (Figure S5). Besides the proteins of the toluene degradation pathway, proteins which were exclusively detectedin anaerobically-grown cells were involved in environmental sensing, flagellum synthesis, transport processes across the membrane, transcription, translation, and folate metabolism.

**Figure 4:**
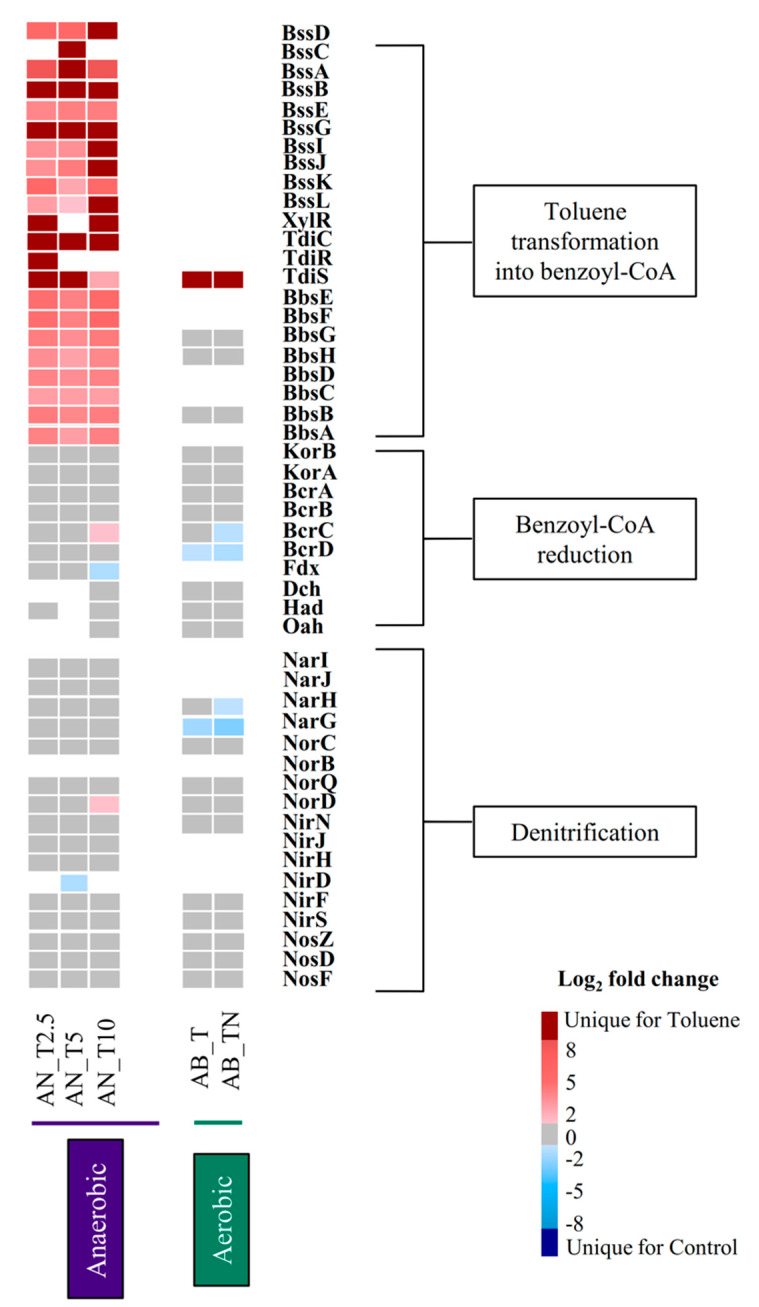
Heatmap visualization of protein regulation required for toluene and benzoate degradation pathways in anaerobic denitrifying and aerobic cultures of *Magnetospirillum* sp. 15-1. Up-regulated proteins are represented by *n* ≥ 1 and are coloured in shades of red. Down-regulated proteins are represented by *n* ≤ −1 and are coloured in shades of blue. AN_T represents cultures growing anaerobically with toluene and either 2.5 mM (AN_T2.5), 5 mM (AN_T5), or 10 mM (AN_T10) of KNO_3_. AB_T represents cultures growing aerobically with benzoate and supplied additionally with either toluene alone (AB_T) or toluene and 5 mM of KNO_3_ (AB_TN). BssD, benzylsuccinate activating enzyme; BssABC, benzylsuccinate synthase; BssE, putative chaperone; BssG-J and BssL, unknown function; BssK, mRNA binding protein; XylR, σ^54^-dependent regulator, TdiC, regulator of TdiR activity; TdiR, toluene degradation regulator; TdiS, toluene degradation inducing sensor; BbsEF, (3-methyl)benzylsuccinate CoA transferase; BbsG, (3-methyl)benzylsuccinyl-CoA dehydrogenase; BbsH, (3-methyl)phenylitaconyl-CoA hydratase; BbsCD, 2-[hydroxyphenyl(methyl)]-succinyl-CoA dehydrogenase; BbsAB, (3-methyl)benzoylsuccinyl-CoA thiolase; BclA, benzoate-CoA ligase; KorBA, 2-oxoglutarate oxidoreductase; BcrABCD, benzoyl-CoA reductase; Fdx, 4Fe-4S ferredoxin; Dch, dienoyl-CoA hydratase; Had, 6-hydroxycyclohex-1-ene-1-carboxyl-CoA dehydrogenase; Oah, 6-oxocyclohex-1-enecarboxyl-CoA hydrolase; NarGHI, nitrate reductase; NarJ protein assembly; NorCB, nitric oxide reductase; NorQ and NorD, unknown function; NirN, haem d1 insertion protein; NirJHLD, unknown functions; NirF, membrane attached lipoprotein; NirS, nitrite reductase; NosZ, nitrous oxide reductase; NosD, putative Cu insertase; NosF, ATP-hydrolyzing of ABC transporters.

Under nitrate-reducing conditions with toluene as growth substrate, all proteins known to be needed for transformation of toluene into benzoyl-CoA, except for BssP, were detected (Figure 4). In comparison with the corresponding benzoate-grown cells, BssA ranked almost always among the proteins with the highest fold change (log_2_-fold range of 5.9 to 8.1, *p*-value < 0.05). The exception was the cultivation with 5 mM nitrate, were no BssA fold change could be calculated because the protein could not be reliably measured in benzoate grown cells. For all other *bss* gene products, the protein abundance was at least 1.7 log_2_-fold higher (range 1.7—6.4 log_2_-fold, *p*-value < 0.05) in toluene-grown versus benzoate-grown cells. The protein abundances of the 5 Bbs enzymes were 2.38 — 5.3 log_2_-fold increased (*p*-values < 0.05) in toluene-grown cells as compared to benzoate-grown cells. Proteins needed for the anaerobic transformation of benzoyl-CoA were detected in cells grown under all anoxic conditions with comparably small differences between toluene-grown and benzoate-grown cells.

Under oxic conditions, no *bss* gene product could be identified. Among the enzymes needed for converting benzylsuccinate into benzoyl-CoA, only BbsB and BbsG were detected at low abundances. In contrast, most proteins of the benzoyl-CoA pathway were found, although their abundances were low (Data set S3). These results matched the observed protein occurrences in the PFRs [27,34]. The substantial *bssA* induction without concomitant detection of the otherwise abundant protein provides strong evidence for post-transcriptional regulation of BSS in strain 15-1 under oxic conditions.

### 3.4. Expression Pattern of the Transcriptional Regulatory Components TdiCRS and XylR

In *Azoarcus* sp. CIB, the Tdi two-component regulatory system controls expression of the *bss* and *bbs* cluster as well as its own genes [16]. In cells of strain 15-1 grown anaerobically on toluene and with 2.5 mM nitrate, TdiC, TdiS, and TdiR were detected. With nitrate concentrations of 5 mM and 10 mM, TdiC and TdiS but not TdiR were found in toluene-grown cells (Figure 4). Only low cellular copy numbers of TdiR are likely to be needed for effective regulation (Leuthner and Heider, 1998), which may explain why the protein was not detected in these samples. TdiC and TdiR were not found in any other condition than during anaerobic growth with toluene, while TdiS was also detected in cells grown anaerobically on benzoate or aerobically in the presence of toluene. These findings indicate that synthesis level of TdiCRS in strain 15-1 depends on the presence of toluene (or benzylsuccinate) and apparently also on anoxic conditions, which in turn might affect expression levels of the *bss* and *bbs* genes. 

The NtrC/XylR-type transcriptional regulator was detected only in cells grown under anoxic conditions on toluene. It remains to be shown whether this protein has any regulatory effect on *bss* and *bbs* gene expression in strain 15-1.

### 3.5. Proteins Involved in the Denitrification Process

Most denitrification protein abundances remained unchanged among the various anaerobic cultures (Figure 4). Only the catalytically active subunit of nitric oxide reductase, NorB, was infrequently detected probably due to its integral membrane protein nature [64]. Under oxic conditions, the majority of proteins involved in denitrification were present too, albeit down-regulated by −0.99 to −2.74 log_2_-fold relative to benzoate-grown cells. The presence of these proteins under oxic conditions matched the previous finding of denitrification occurring in the PFRs [27].

## 4. Discussion

For microbes capable of anaerobic toluene degradation and living in habitats in which anoxic and oxic conditions frequently alternate, the regulatory decision to synthesize BSS is not trivial [13]. The active enzyme is rapidly and irreversibly destroyed by molecular oxygen. Even if a radical quenching mechanism of the active form into the inactive one by, e.g., a small diffusible molecule would be in place [65,66], it is unlikely that such a mechanism would be faster at all times than molecular oxygen reaching the radical site of BSS. In addition, there is evidence for oxygen sensitivity of BssB and BssC [14,15]. Furthermore, BSS is a kinetically slow enzyme. In *Thauera aromatica* growing on toluene it makes up several percent of the soluble protein content of the cell [67]. Thus, the timing and strength of BSS synthesis would ideally be tuned according to the dynamics of the ambient conditions in order to prevent that the cell is burdened with too much unusable or even destroyed enzyme. A respective regulatory scheme would need to process multiple sensory inputs from environmental and cellular clues. 

In this study we present novel insights into the complex regulation of BSS expression in *Magnetospirillum* sp. strain 15-1. Firstly, and the key finding of this study, the synthesis of BSS is under post-transcriptional regulation in this strain. This novel type of regulation for the GRE family constitutes a bacterial decision gateway, allowing for a quick adaptation process to conditions in the ambient ecosystem that are amenable for anaerobic toluene degradation. Post-transcriptional regulators can be RNA binding proteins (RBP), ribonucleases, helicases, or small RNA molecules [68,69,70,71], all of which can modulate mRNA stability or the initiation of translation. They may act on a global transcriptome level or be restricted to a small set of transcripts. We assume that the stringently controlled translation of the *bss* gene products is governed by a specific regulatory scheme rather than by a global translational regulator involved in the transition between aerobic and anaerobic growth. A candidate regulator is BssK, which is encoded in the *bss* gene cluster in all sequenced denitrifying and some sulfate-reducing bacteria capable of anaerobic toluene degradation. BssK is a member of the short-chain dehydrogenases/reductases (SDR) superfamily [72]. The widely distributed SDR protein fold is adaptable to diverse functions including mRNA binding and RNase activity [73,74]. BssK belongs to the atypical subgroup 1, which members lack the canonical active site residues of SDR enzymes with dehydrogenase/reductase function [72]. Some of the subgroup 1 proteins are postulated to be involved in redox sensing [75]. The detection of BssK exclusively under anoxic conditions suggests that this protein acts as a translational activator in the absence of oxygen. Considering that this strain´s capability to degrade toluene anaerobically is likely the result of horizontal gene transfer, this regulatory feature could be common among denitrifying toluene degraders. 

Secondly, the *bss* genes are transcribed already under oxic conditions in strain 15-1, and the transcription increases further in the absence of oxygen. The expression of *bssA* under oxic conditions is congruent with its comparably high expression level in the PFRs [27,34]. Similarly, in the facultative anaerobe *Thauera* sp. strain DNT-1 approximately equal levels of *bssA* transcripts were detected during aerobic and anaerobic growth, both with toluene as the sole carbon source [24]. These findings are in contrast to the regulatory pattern in *Azoarcus* sp. strain CIB and *Magnetospirillum* sp. strain TS-6, in which *bssA* was not transcribed under oxic conditions in the presence of toluene [16,23]. To date, there is no sequence information available for regulator genes in strains DNT-1 and TS-6, which hampers the formulation of convincing mechanistic and eco-physiological explanations for these differences even among closely related strains. One hypothesis we would like to offer is that there are TdiS varieties which differ in their responses to toluene and benzylsuccinate: the “strain CIB type”, which responds (predominantly) to benzylsuccinate [16], and the “strain 15-1 type” for which toluene is the principle effector. Still, this picture may not be complete since in strain CIB there was Tdi-independent activity of the *bss* promoter in the presence of toluene [16], and in strain 15-1 further components such as the XylR-like protein could play a role in *bss* expression, too.

Thirdly, and so-far unique to strain 15-1, the Tdi system encompasses the TdiC module. According to sequence analysis, this module may be involved in catabolite repression via sensing the cellular energy status. In strain CIB there is evidence that catabolite repression is co-regulating expression of the *bss* and *bbs* genes, yet no regulatory element is known that could modulate activity of the Tdi two-component system in that strain [16]. Catabolite repression was also reported for various strains carrying out aerobic toluene degradation [76,77]. Such a regulatory feature would provide an obvious advantage for toluene-degrading microbes such as strain 15-1 that are catabolically versatile and thrive in habitats with comparable high fluxes of labile organic carbon.

Fourthly, there was no indication that nitrate needed to be present for transcribing the *bss* genes, which was suggested to be of importance for toluene-degrading denitrifiers [25]. In contrast, *bssA* transcription declined in cells grown anaerobically with toluene when the nitrate concentration was raised to 10 mM. It appears that this decline was specific since *bcrC* transcription was not affected. We hypothesize that comparable high concentrations of denitrification intermediate(s) repress *bssA* transcription. Nitric oxide (NO) has the highest chemical reactivity among the free intermediates and as a free radical NO has a strong propensity to interact with GREs. Although there is to our knowledge no biochemical data published on NO sensitivity of BSS, we assume that it is already inhibitory at nM concentrations. We further hypothesize that during denitrification the presence of high concentrations of nitrate resulted in a slightly increased steady-state level of NO, and that this increase, although minute, did have a measurable effect on *bss* expression. There was also a decrease in *bssA* expression in cells grown under oxic conditions after the addition of nitrate. In this study, we did not attempt to quantify denitrification rates under oxic conditions; however, this was described to occur in various other bacteria [78,79], and denitrification did apparently take place in the oxic PFRs. The genome of strain 15-1 harbors eight members of the CRP/FNR superfamily with closest similarities to NO sensors/regulators, five of which were detected in cells grown under oxic and anoxic conditions (Figure S6). Such regulators control transcription of various genes including some involved in degradation of aromatic compounds [80], denitrification, oxidative stress response [60] and transcriptional regulation [81]. For instance, FnrL mediates transcriptional repression of members of the *luxR* family [81], to which *tdiR* belongs [19]. This type of repression could negatively affect BSS transcription in strain 15-1. 

In conclusion, the regulatory scheme for BSS expression can be more complex than appreciated up to now. In *Magnetospirillum* sp. strain 15-1 thriving in the rhizosphere of a constructed wetland model, post-transcriptional control of BSS synthesis might endow the cells with an additional layer of regulation to cope better with their redox-dynamic environment. Furthermore, the metabolically versatile strain 15-1 appears to process various sensory inputs by other regulatory components in addition to the established TdiRS system to control the level of *bss* and *bbs* transcription. Future information on regulation of BSS synthesis will allow for an improved assessment of anaerobic toluene degradation at contaminated sites.

## Figures and Tables

**Table 1:** Primers used in this study.

**Primers Used for PCR**				
**Target Sequence**	**Primer Name**	**Primer Sequence**	**Product Size (bp)**	**Source**
16S rRNA	27F 1492R	AGAGTTTGATCMTGGCTCAG GGYTACCTTGTTACGACTT	1465	[31]
*bssA*	bssA_F1 bssA_R2	GACGARTTCATCRTCGGCTACCACGC AGCAGRTTGSCYTTCTGRTTYTTCTG	1546	Junca, H.
*bcrC*	bcrC_F bcrC_R	CGHATYCCRCGSTCGACCATCG CGGATCGGCTGCATCTGGCC	800	[32]
*bbsC* region	bbsD_F bbsB_R	GGCGGGATGTTGTCCTATGG GCTTCGGCCCTATTTGCTTG	1823	this study
**Primers Used for RT-qPCR**				
**Target Sequence**	**Primer Name**	**Primer Sequence**	**Product Size (bp)**	**Source**
16S rRNA	16S_F 16S_R	TGATGAAGGCCTTAGGGTTG CCAGGGCTTTCACTTCTGAC	170	Marín, V.
*bcrC*	2bcrC_F 2bcrC_R	CATGATCTTCCCGTT TCCTTCAGCTCCTTC	159	Marín, V.
*bssA*	bssA_F3 bssA_R3	CGTCCTTCGCCTCGGGTTAC CATCGCCTGCCAGTTGTCAATC	188	Marín, V.
**Primers Used for RT-PCR**				
**Target Sequence**	**Primer Name**	**Primer Sequence**	**Product Size (bp)**	**Source**
Intergenic region *bssD-bssA*	bssD-bssA_F bssD-bssA_R	CGCATTCACATCCCGGTCATC CAGGACGTTGGCGGTCATATT	575	this study
Intergenic region *bssA-bssE*	bssA-bssE_F bssA-bssE_R	CTGAATTGCGACCTCTGAGC GCTTGCGCGAGATATTACGG	587	this study
Intergenic region *bssE-bssF*	bssE-bssF_F bssE-bssF_R	GGCAGGTCTGGATGGATGAG CGTTTGGGAGGGGTATCGTC	504	this study
Intergenic region *bssF-bssG*	bssF-bssG_F bssF-bssG_R	GCCATTCGCCGTTTCAACAA GCGAACCTGGGAAAACATCG	482	this study
Intergenic region *bssG-bssP*	bssG-bssP_F bssG-bssP_R	CCTTGGATGAAGGCCGTAAC CCGAAGATCAGCCACTTTCC	560	this study
Intergenic region *bssP-bssI*	bssP-bssI_F bssP-bssI_R	CCCGTTTCAAGCGATGGTTC GAACTCCCTCCTTGCGGTAG	493	this study
Intergenic region *bssI-bssJ*	bssI-bssJ_F1 bssI-bssJ_R1	CGAATTCCCCAGCGACTCA ATCTCGAACACGCCCACTC	561	this study
Intergenic region *bssJ-bssK*	bssJ-bssK_F bssJ-bssK_R	GCCCCCTACTTCAAATGGCT GTATCCCAATCCAGCGGAGG	480	this study
Intergenic region *bssK-bssL*	bssK-bssL_F bssK-bssL_R	AGTTGATTTCCTACGGGCGG GTGAGCAATCCACATGACGC	515	this study
Intergenic region *tdiC-tdiR*	tdiC-tdiR_F tdiC-tdiR_R	GAGATCATGACGACGGAGGT TAGAAAGATCAGCGGCAGCG	500	this study
Intergenic region *tdiR-tdiS*	tdiR-tdiS_F tdiR-tdiS_R	CGCCTCAGCAAGGAAGTGTT GGAAGCAAATGCCAACGGG	435	this study
*xylR*	xylR_F xylR_R	TGTCGAGCGTGGCTATTACTC CTTCCACCAAATTCTCGGGC	545	this study
Intergenic region *bbsA-bbsB*	bbsA-bbsB_F bbsA-bbsB_R	GGGCAGCTTGATTTTCCCAA GGCGATGAACACATCTCGTTG	436	this study
Intergenic region *bbsB-bbsC*	bbsB-bbsC_F bbsB-bbsC_R	GGCGGGATGTTGTCCTATGG ACCGATTCCGGAAGGAAAGG	535	this study
Intergenic region *bbsC-bbsD*	bbsC-bbsD_F bbsC-bbsD_R	CGGCAAGAGCGCCTATTTCT GGTGATTCCATTGCGTCCCA	611	this study
Intergenic region *bbsD-bbsE*	bbsD-bbsE_F bbsD-bbsE_R	CTGGGACGCAATGGAATCAC GCGCAAAAACACCTCCCTG	542	this study
Intergenic region *bbsE-bbsF*	bbsE-bbsF_F1 bbsE-bbsF_R1	ATACGCCTATCGGACCTCGG GCTCTGCATGAACCACTTCC	380	this study
Intergenic region *bbsF-bbsG*	bbsF-bbsG_F bbsF-bbsG_R	CGCGGTGTTTTCCGATGAAG TTCAGCATTGCGTGCTCTTG	550	this study
Intergenic region *bbsG-bbsH*	bbsG-bbsH_F bbsG-bbsH_R	CGGGAAACCGGCATCGAATA TTCATCTGGGGGATGAGGGG	428	this study

## Supplementary Materials

The following are available online at https://www.mdpi.com/2076-2607/8/5/681/s1, Figure S1: Growth of *Magnetospirillum* sp. 15-1 at different conditions, Figure S2: Pairwise alignment of TdiS and TdiR proteins among anaerobic toluene degraders, Figure S3: Transcriptional organization of *bss*, *tdi*, and *bbs* genes, Figure S4: Fold change averages of *bcrC* expression in anaerobic and aerobic cultures of *Magnetospirillum* sp 15-1, Figure S5: Metabolic pathways detected in anaerobic and aerobic cultures of *Magnetospirillum* sp. 15-1, Figure S6: Phylogenetic tree of CRP/FNR regulators.Table S1: Identity (%) of proteins involved in anaerobic toluene degradation among different degraders to *Magnetospirillum* sp. 15-1, Table S2: Detection of CRP/FNR superfamily members, Table S3: Culture pairs used to assess *bssA* and *bcrC* expression from toluene-grown cells relative to *bssA* and *bcrC* expression from benzoate-grown cells. Data set S1: qPCR raw data of *bssA*, *bcrC* and 16S rRNA from AN and AB cultures, Data set S2: Copy numbers of *bssA*, *bcrC* and 16S rRNA from AN and AB cultures, Data set S3: Proteins identification and abundances from AN and AB cultures.
