# Supplementary material for: Benzylsuccinate Synthase is Post-Transcriptionally Regulated in the Toluene-Degrading Denitrifier Magnetospirillum sp. Strain 15-1
Source: Microorganisms. 2020 May 7;8(5):681. doi: 10.3390/microorganisms8050681 (PMC7285207; doi:10.3390/microorganisms8050681)
Supplement: Supplementary file 1 [file microorganisms-08-00681-s001.pdf]

Article

Benzylsuccinate synthase is post-transcriptionally regulated in the toluene-degrading denitrifier *Magnetospirillum* sp. strain 15-1

Ingrid Meyer-Cifuentes[1,2], Sylvie Gruhl[1], Sven-Bastiaan Haange[3], Vanessa Lünsmann[3], Nico Jehmlich[3], Martin von Bergen[3,4], Hermann J. Heipieper[1*], Jochen A. Müller[1]

1 Department of Environmental Biotechnology, Helmholtz Centre for Environmental Research - UFZ, Permoserstr. 15, 04318 Leipzig, Germany; ingrid.meyer.cifuentes@dsmz.de (I.M.-C.) sylvie.gruhl@outlook.de (S.G.), jochen.mueller@ufz.de (J.A.M)

2 Junior Research Group of Microbial Biotechnology, Leibniz Institute DSMZ, German Collection of Microorganisms and Cell Cultures, Inhoffenstr. 7B, 38124 Braunschweig, Germany

3 Department of Molecular Systems Biology Helmholtz Centre for Environmental Research - UFZ, Permoserstr. 15, 04318 Leipzig, Germany; sven.haange@ufz.de (S.-B.H.), vanessa.luensmann@posteo.de (V.L.), nico.jehmlich@ufz.de (N.J.)

4 Group of Functional Proteomics, Institute of Biochemistry, Faculty of Biosciences, Pharmacy and Psychology University of Leipzig, Talstrastr. 33, 04103 Leipzig, Germany; martin.vonbergen@ufz.de

* Correspondence: hermann.heipieper@ufz.de; Tel.: +49-341-2351694.

Keywords: toluene, nitrate, benzylsuccinate synthase, *Magnetospirillum*, rhizosphere

1. Supplemental Figures and Tables

(a) **(a.1)**

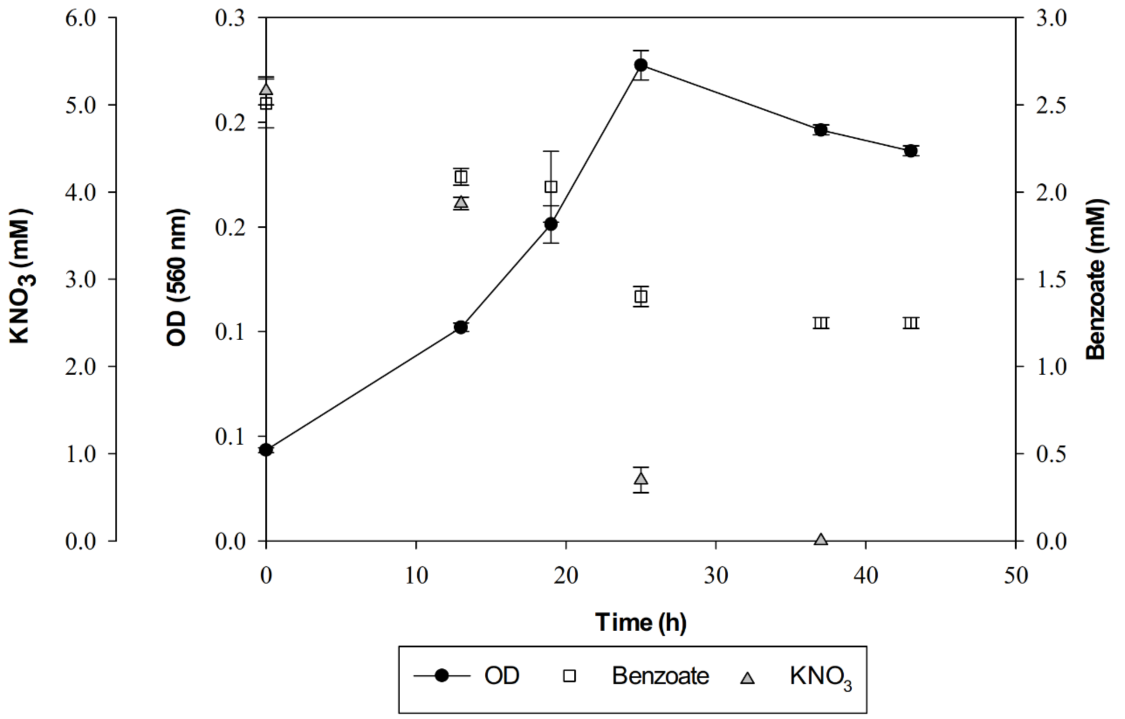

(a.2)

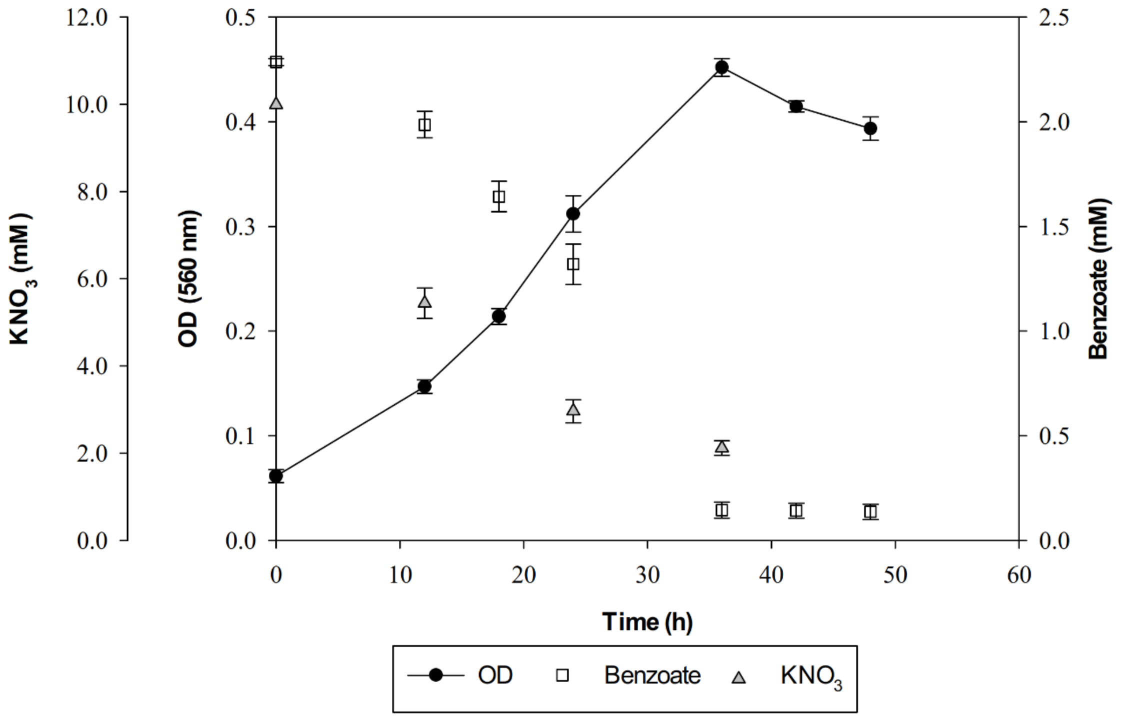

(b)

(b.1)

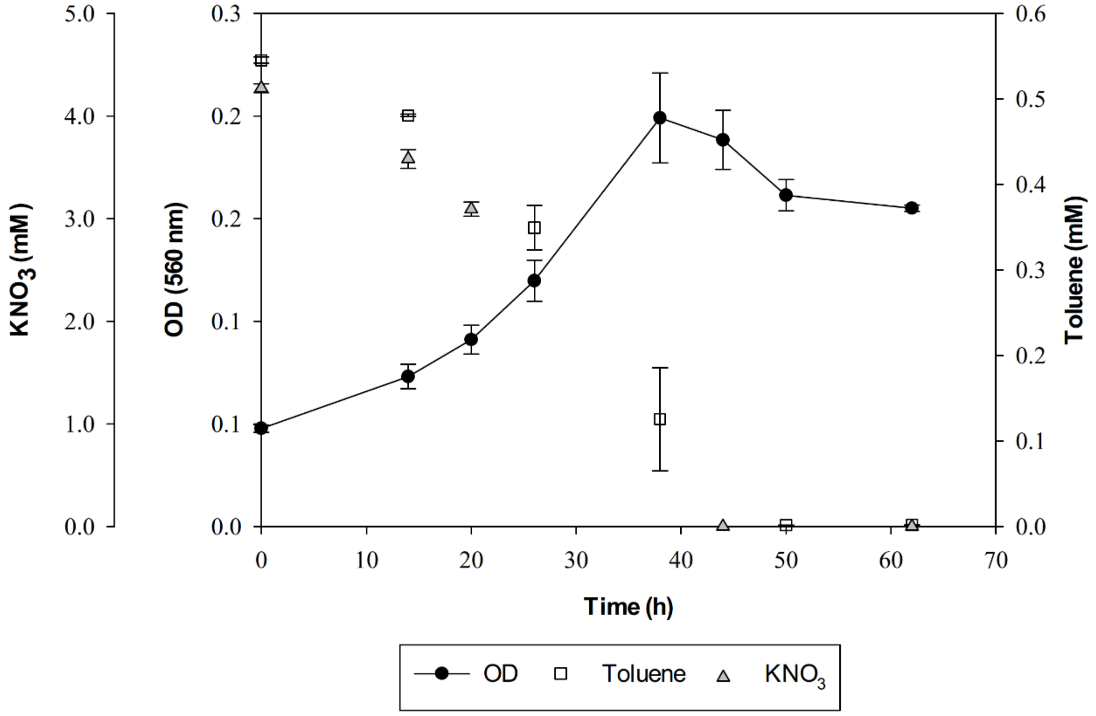
KNO3 (mM)
5.0
4.0
3.0
2.0
1.0
0.0
OD (560 nm)
0.3
0.2
0.2
0.1
0.1
0.0
Toluene (mM)
0.6
0.5
0.4
0.3
0.2
0.1
0.0
0
10
20
30
40
50
60
70
Time (h)
OD
Toluene
KNO3

(b.2)

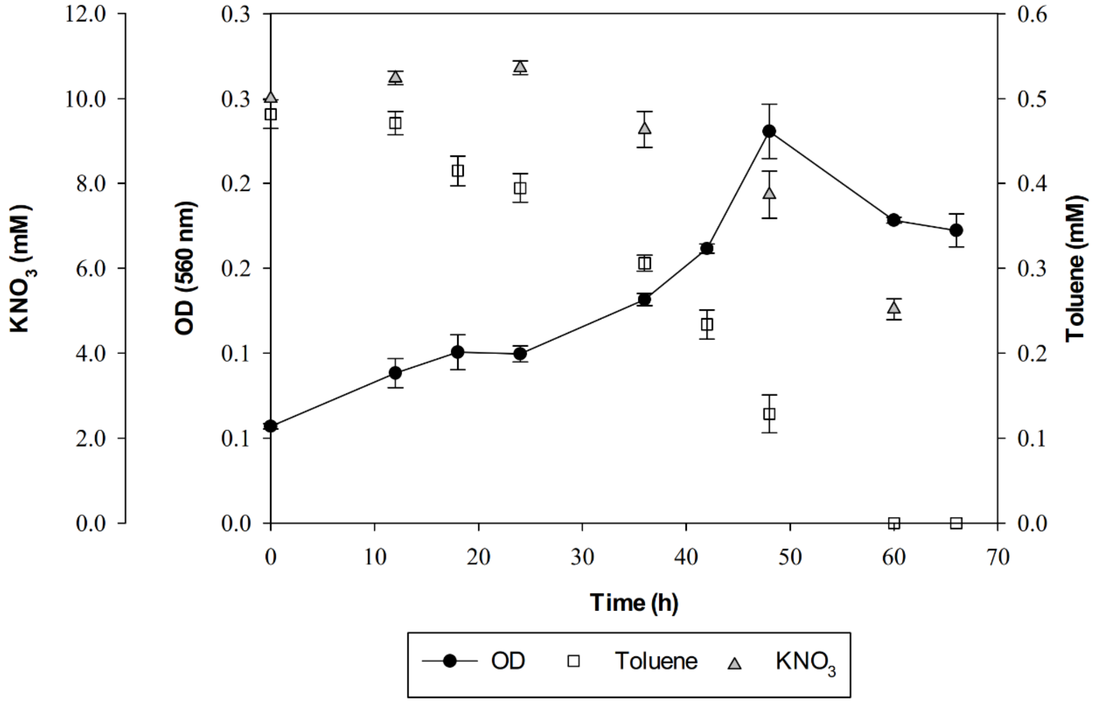
KNO3 (mM)
12.0
10.0
8.0
6.0
4.0
2.0
0.0
OD (560 nm)
0.3
0.3
0.2
0.2
0.1
0.1
0.0
Toluene (mM)
0.6
0.5
0.4
0.3
0.2
0.1
0.0
0
10
20
30
40
50
60
70
Time (h)
OD
Toluene
KNO3

(c)

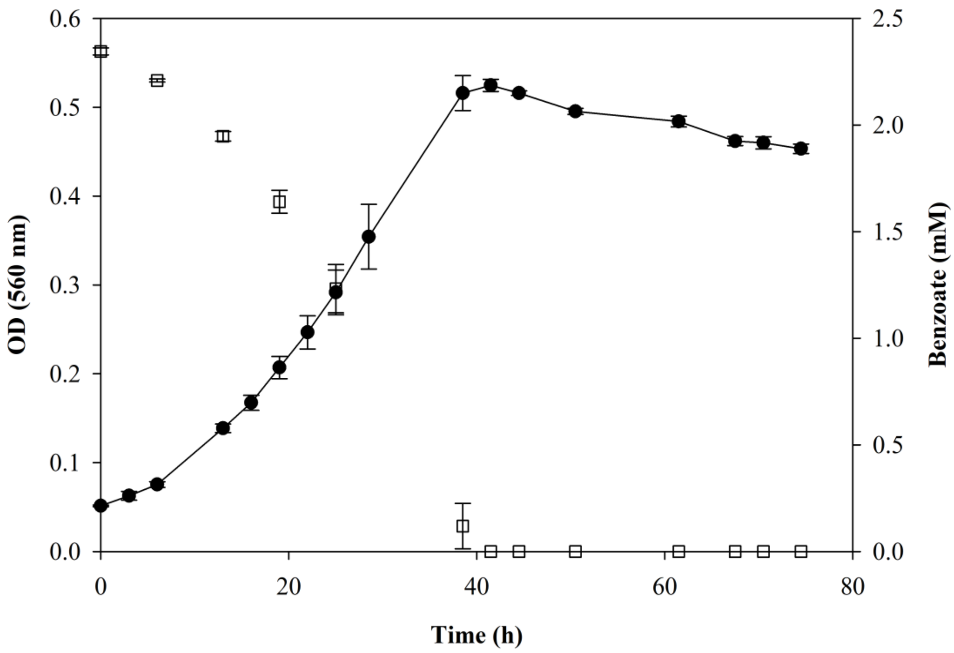

Figure S1: Growth of *Magnetospirillum* sp. 15-1 at different conditions. Growth of *Magnetospirillum* sp. 15-1 with: (a) 2.5 mM benzoate as the sole carbon source, 5 mM (a.1) and 10 mM KNO3 (a.2) under anoxic conditions; (b) 0.5 mM toluene as the sole carbon source, 5 mM (b.1) and 10 mM KNO3 (b.2) under anoxic conditions; (c) 2.5 mM benzoate as the sole carbon source under oxic conditions. Nitrite was below detection limits in each culture. Δ: Nitrate consumption; ●: optical density; □: carbon source degradation.

microorganisms

MDPI

Table S1: Identity (%) of proteins involved in anaerobic toluene degradation among different degraders to *Magnetospirillum* sp. 15-1[a].

**Protein**	***Magnetospirillum* sp. TS-6 AB167725.1**	***Azoarcus tolulyticus* ATCC 51758 NZFTMD01000027.1**	***Azoarcus toluclasticus* ATCC 700605 ARJX01.1**	***Azoarcus* sp. CIB DQ988527.1**	***Azoarcus* sp. EbN1 NC006513**	***Thauera aromatica* K172 CP028339.1**	***Herminiimonas* sp. CN AVCC00000000.1**	***Desulfobacula toluolica* Tol2 NC018645**	***Geobacter metallireducens* GS-15 CP000148**
**BssD**	98.8	65.6	64.7	65.6	62.8	62.2	63.1	57.5	48.2
**BssC**	98.3	62.7	61	61	63.8	62.1	58.6	55.2	56.9
**BssA**	99.8	83.9	83.6	82.5	81.8	80.6	85.5	76.2	74.1
**BssB**	100	69	72.3	68.7	73.3	74.7	65.5	53.6	56.3
**BssE**	98.6	79.2	79.2	79.2	81.7	79.6	82.1	67.7	64.5
**BssF**		60.7	59.6	61.3	61.4	63.6	63	51.9	48.1
**BssG**		51.6	51.6	51	52.9	51.8	54.7		
**BssI**		50.8		50.3			47.7		
**BssJ**		64.9	61.7	64.9	63.6	63	58.9		
**BssK**		54.8	69.1	54.8	56.7	58.9	55		
**BssL**		48.5	48.7	49	48.7	50.1	51.7		
**XylR**									41.8

**TdiR**		63.5	63.5	63.2	59.8	65.2		
**TdiS**	52.3		58.3	58.9	58.5	58.9		
**Ycf48**		34						
**YdfJ**		49.5						
**BbsH**	82.1	81.3	79.4	79.8	80.2	79.8	72	72
**BbsG**	77.6	77.1	77.4	78.4	77.4	75.2	69.9	71.6
**BbsF**	73.2	74.6	73.7	74.4	74.6	75.6	67.5	71
**BbsE**	69.7	71.8	69.4	70.6	70.4	72.8	63.3	64.9
**BbsD**	74.3	72.3	73.9	73.9	73.9	75.1	67.1	68.3
**BbsC**	44	43.7	44.8	44	44	45.2	42.9	39.6
**BbsB**	84.4	84.6	84.6	85.4	85.6	83.1	73.1	74.4
**BbsA**	74.5	74.5	73.8	71.1	74.5	72.8	61.8	66.2

[a] ClustalW 2.1 alignment; Cost Matrix: BLOSUM62; Gap open cost:10; Gap extend cost: 0.1

microorganisms

MDPI

(a)

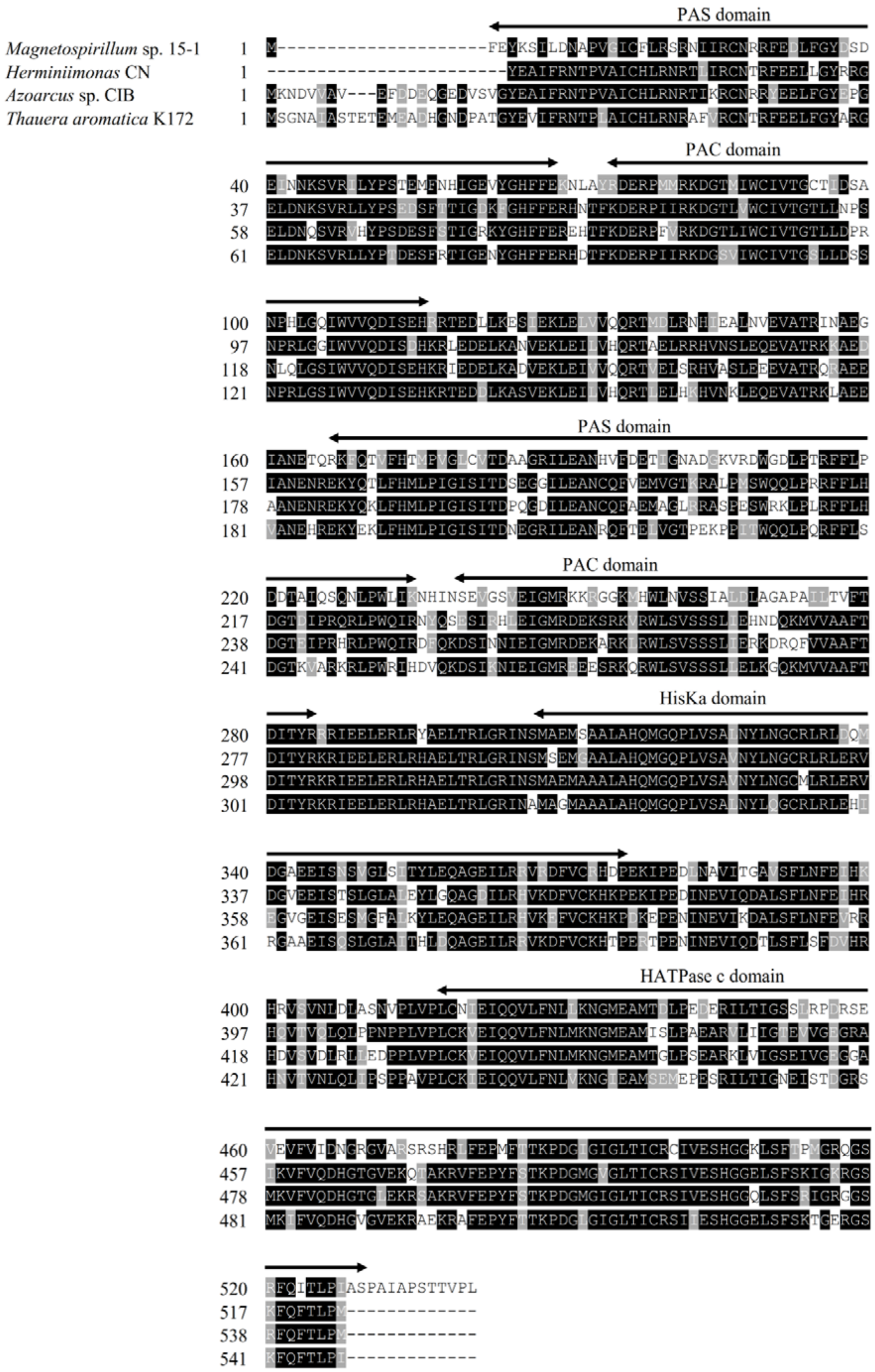

(b)

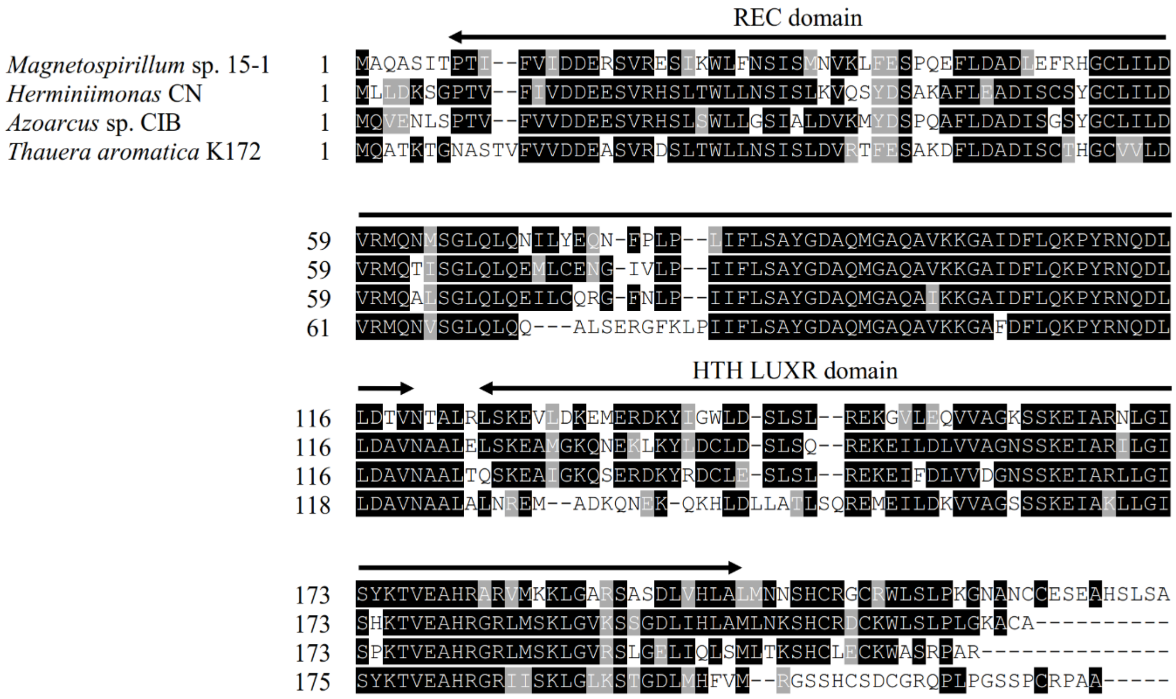

Figure S2: Pairwise alignment of TdiS and TdiR proteins among anaerobic toluene degraders. The alignment of TdiS (a) and TdiR (b) are shown for four different toluene degraders. Shaded background represents the similarity of the aminoacidic residue between the different aminoacidic sequences. Arrows placed above the first aminoacidic sequence highlights conserved protein domain.

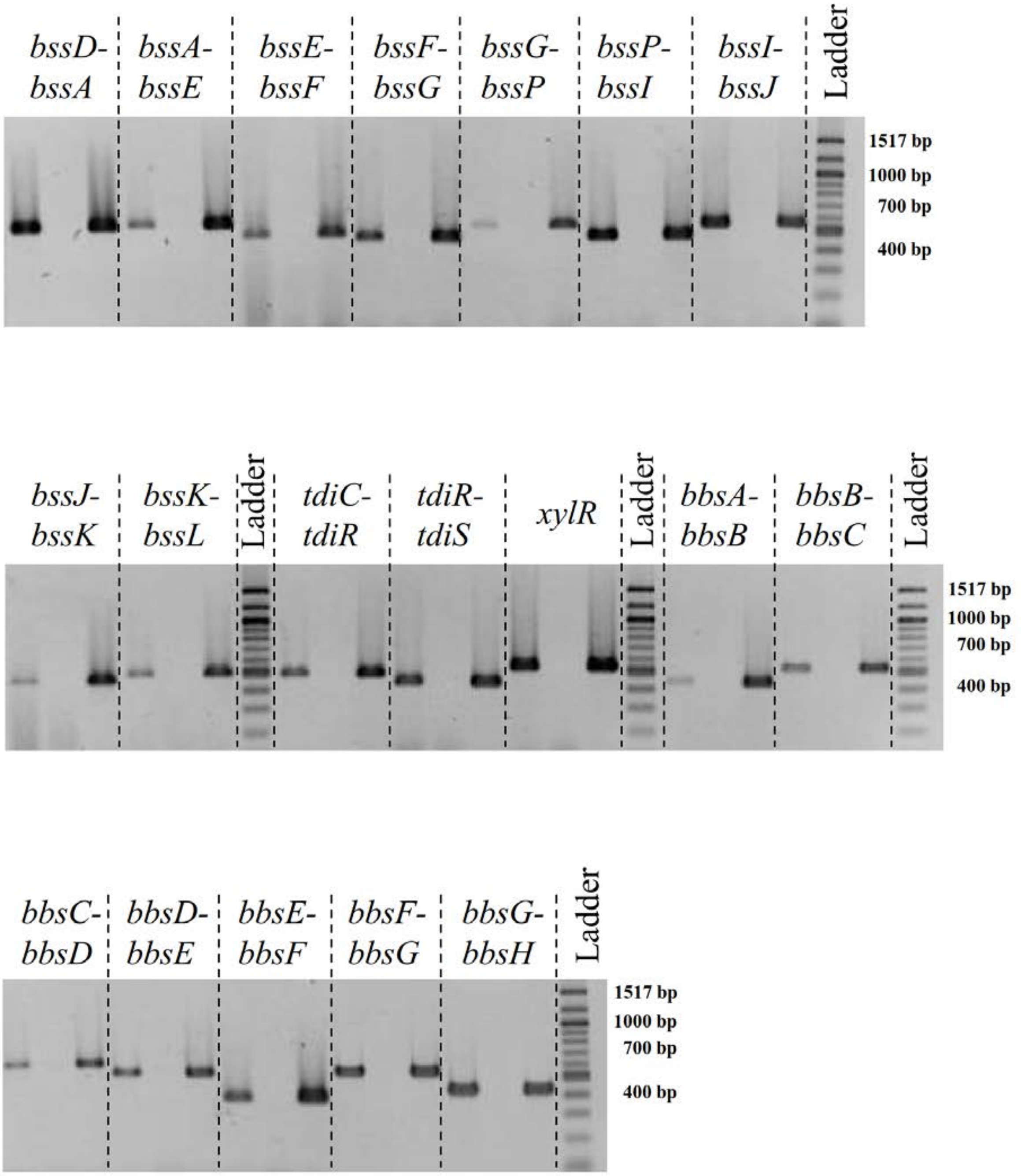

Figure S3: Transcriptional organization of *bss*, *tdi*, and *bbs* genes. The three lanes per target sequence region show results of RT-PCR assays with: From left to right, (i) RNA as template and with RT, (ii) with RNA as template but without RT (negative control), and (iii) with genomic DNA (positive control).

Data Set S1: qPCR raw data of *bssA*, *bcrC* and 16S rRNA from AN and AB cultures.

Data Set S2: Copy numbers of *bssA*, *bcrC* and 16S rRNA from AN and AB cultures.

Table S2: Detection of CRP/FNR superfamily members. Regulation of each protein is indicated (log2 fold) and their significance (p-value) in anaerobic and aerobic cultures. AN_T represents cultures growing anaerobically with toluene and either 2.5 mM (AN_T2.5), 5 mM (AN_T5), or 10 mM (AN_T10) of KNO_3. AB_T represents cultures growing aerobically with benzoate and supplied additionally with either toluene alone (AB_T) or toluene and 5 mM of KNO_3 (AB_TN).

		Anaerobic cultures						Aerobic cultures			
**Gene**	**Locus**	**log2 AN_T2.5**	**p-value AN_T2.5**	**log2 AN_T5**	**p-value AN_T5**	**log2 AN_T10**	**p-value AN_T10**	**log2 AB_T**	**p-value AB_T**	**log 2 AB_TN**	**p-value AB_TN**
**contig01_gene37**	39763-40416	ND	ND	ND	ND	ND	ND	ND	ND	ND	ND
**contig01_gene283**	296369-295611	**-0.123**	**0.797**	**-1.353**	**0.172**	**-1.213**	**0.099**	ND	ND	ND	ND
**contig01_gene551**	568946-569650	**-0.861**	**0.022**	**-0.331**	NC	**-0.178**	**0.724**	ND	ND	ND	ND
**contig04_gene2311**	291845-292429	ND	ND	ND	ND	ND	ND	ND	ND	ND	ND
**contig04_gene2534**	518994-519695	**-0.307**	**0.358**	**-0.344**	**0.588**	**-0.323**	**0.211**	**-0.230**	**0.604**	**0.611**	**0.643**
**contig06_gene3113**	217618-218352	ND	ND	ND	ND	ND	ND	ND	ND	ND	ND
**contig07_gene3349**	117592-116894	**-0.048**	**0.847**	**0.065**	**0.724**	**-0.226**	**0.439**	**0.355**	**0.642**	**0.882**	**0.135**
**contig07_gene3350**	118295-117597	**-0.056**	**0.900**	**-0.231**	**0.267**	**-1.105**	**0.028**	**0.054**	NC	**0.088**	NC
**contig09_gene3888**	164717-165376	ND	ND	ND	ND	NC	NC	ND	ND	ND	ND
**contig09_gene3893**	170577-169882	**-0.410**	**0.612**	**-0.953**	NC	**-1.182**	**0.050**	ND	ND	ND	ND
**contig10_gene4043**	99521-96201	**-1.371**	NC	**-0.788**	NC	**0.605**	NC	**0.206**	NC	**0.558**	NC
**contig10_gene4044**	96209-97012	**2.421**	**0.073**	ND	ND	**-1.268**	NC	**-0.381**	NC	**-0.471**	NC

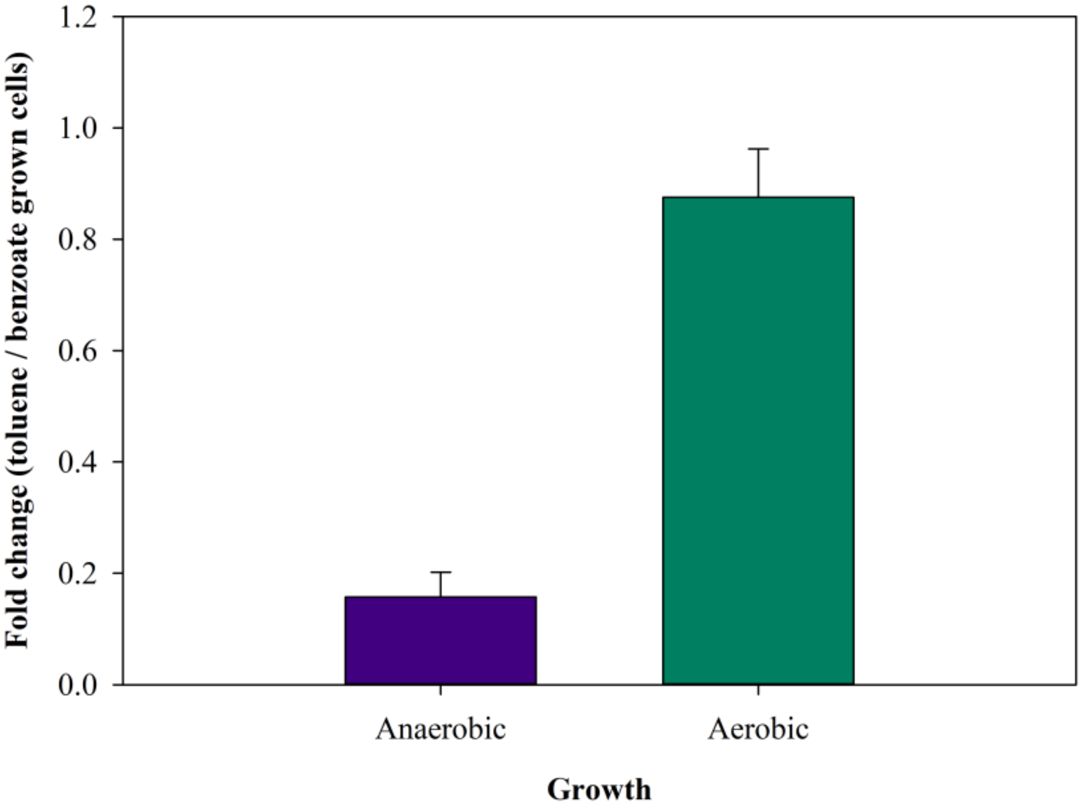

Figure S4: Fold change averages of *bcrC* expression in anaerobic and aerobic cultures of *Magnetospirillum* sp 15-1. AN cultures growing with 2.5 mM, 5 mM, or 10 mM of KNO_3 are shown as "Anaerobic" and AB cultures growing in the presence of toluene and toluene/nitrate are shown as "Aerobic".

Data Set S3: Proteins identification and abundances from AN and AB cultures.

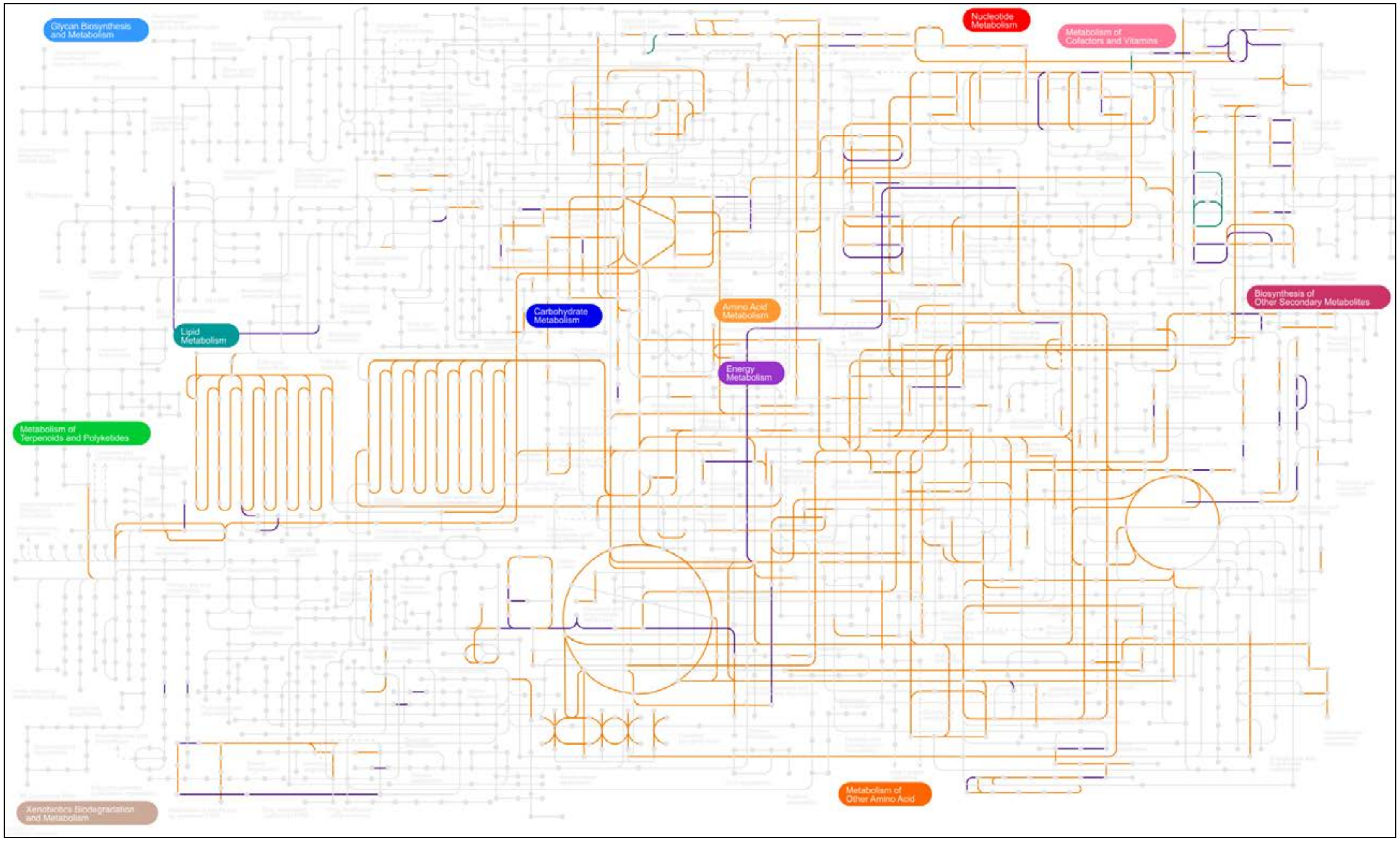

Figure S5: Metabolic pathways detected in anaerobic and aerobic cultures of *Magnetospirillum* sp. 15-1. KEGG pathways mapping shows pathways unique to AN cells (purple), unique to AB cells (green), and shared between both conditions (orange).

microorganisms MDPI

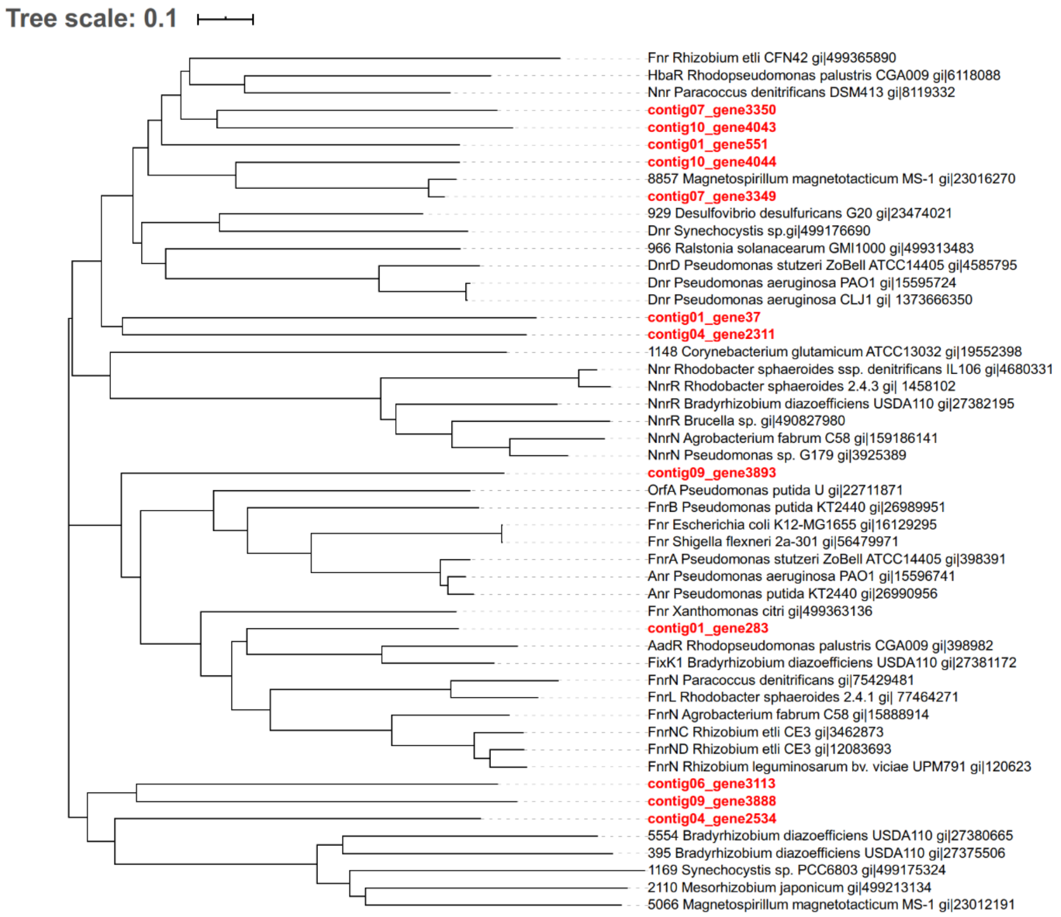

Figure S6: Phylogenetic tree of CRP/FNR regulators. Aminoacidic sequences were obtained from the genome of *Magnetospirillum* sp. 15-1 (shown in red) and other amino acid sequences from the NCBI database. Dnr, Fnr, Nnr and C represent branches of the family and their inducers and/or repressors are shown (NO, O_2). The phylogenetic relationship was conducted with the Neighbor-Joining method (1000 bootstrap replicates). The tree scale represents amino acids substitution per site.

2. Supplemental Materials and Methods

2.1. Benzoate detection

Degradation of benzoate was quantified through a gradient HPLC with a UV detection of 228 nm. Benzoate was separated from other compounds with a LiChrospher 100 RP-18e (5 µm), LiChroCART 125-4, column. Methanol at 100 % (Rotisolv HPLC Gradient Grade 99 %, Carl Roth, Karlsruhe, BW, Germany) and phosphoric acid at 0.1 % (Chemsolute for analysis 85 %, Th. Geyer, Höxter, NW, Germany) were used as eluents of the mobile phase. Methanol was increased from 20 to 100% until minute 20 and remained constant for 5 min.Then methanol was decreased again to 20% and remained constant for 3 min. The flow rate was set at 0.5 mL/ min. Quantification was performed by using calibration curves.

2.2. ***RNA quality and purity analysis***

To assess the quality of total RNA from each elute, 1 µL of sample was measured by Nanodrop (Nanodrop ND-1000 Spectrophotometer, Thermo Fisher Scientific, Waltham, MA, USA) to determine purity by 260/230 ratios and to check contaminants such as guanidine salts and phenol. To analyse RNA quality, 5 µL of each RNA elute was run in a 1.2 % agarose gel for 45 min at 90 v.

To test for DNA residuals within the samples, 1 µL of each elute was subjected to PCR amplification with a HotStar Taq ® according to manufacturer's instructions and by using the universal primers 27F and 1492R. Then, 5 µL of each amplicon was run in a 1.2 % agarose gel for 30 min at 100 V.

2.3. ***cDNA synthesis***

Total RNA (150 ng) was used to synthesize cDNA through reverse transcriptase technique using the Ominiscript® Reverse Transcription Kit (Qiagen, Hilden, NW, Germany). First, the RNA samples were dissolved each in RNAse free-water to make 12.75 µL and incubated for 10 min at 65 °C to denature RNA secondary structure. Then a master mix was prepared and each reaction consisted of 2 µL 10x buffer RT, 2 µL dNTP mix (5 mM each), 2 µL random hexamer primers (10 µM), 1 µL RNase inhibitor (10 units/ µL) and 1 µL Ominiscript Reverse Transcriptase. Negative controls without the reverse transcriptase (NRTs) enzyme were prepared per each sample. Each reaction had a final volume of 20 µL. cDNA synthesis was performed by incubating the samples at 37 °C for 60 min. Samples were then stored at -20 °C until usage.

2.4. ***Preparation of the bssA, bcrC and 16S rRNA standards for qPCR***

The genes *bssA*, *bcrC* and 16S rRNA were amplified by PCR from extracted DNA of 15-1 cells growing exponentially with toluene. The primer pairs used for amplification are shown in Table 1. The PCR reaction consisted of 12.5 µL HotStarTaq Master Mix 2x (Qiagen, Hilden, NW, Germany), 1 µL of each primer (0.4 µM final concentration), 9.5 µL of RNase free-water and 1 µL template. The amplicons were further run in an 1.2% agarose gel and subsequently purified using the Qiagen QIAquick PCR purification kit (Qiagen, Hilden, NW, Germany) according to the manufacturer´s instructions. The concentration of the purified DNA was measured with a Nanodrop (Nanodrop ND-1000 Spectrophotometer, Thermo Fisher Scientific, Waltham, MA, USA). Based on the concentration, the number of gene copies per µL was calculated with the following equation (eq.1):

$$\frac{Copies}{\mu L} = \frac{\text{PCR product}\left(\frac{\text{ng}}{\mu\text{L}}\right)\cdot 10e^{-9}}{\left(\text{Length PCR product [bp]}\cdot 660\left[\frac{\text{Da}}{\text{bp}}\right]\right)\cdot 6.022\cdot 10\text{e}^{23}} \quad \text{(eq. 1)}$$

660 Da/bp: Mass of double- stranded DNA; 6.022 • $10e^{23}$: Avogadro constant

2.5. ***qPCR reaction for the amplification of bssA, bcrC and 16S rRNA***

The calibration curve for each gene was prepare by diluting serially the standards with RNase free-water (Qiagen, Hilden, NW, Germany) from 10^8 to 10^1 copies/ µL. cDNA templates were undiluted or diluted 1:10 for aerobic and anaerobic experiments respectively. cDNA templates for 16S rRNA amplification were always diluted 1:10. Negative controls and qPCR blanks were not diluted. To avoid degradation, the samples were kept on ice all time.

A PCR reaction consisted of: 6.25 µL KAPA SYBR FAST, 4.75 µL Rnase free-water, 0.25 µL of each primer (Table 1) with a final concentration of 200 nM for the 16S_F/16S_R primer pairs and 500 nM for bssA_F3/bssA_R3 and 2bcrC_F/2bcrC_R primer pairs. For the amplification of *bssA* and *bcrC* the amount of cDNA template used was 1.5 µL and for 16S rRNA, 1.0 µL. Amplification was performed in a Step One Plus Real Time PCR System (Applied Biosystems, Thermo Fischer Scientific, Waltham, MA, USA).

2.6. *Gene expression analysis*

The initial abundances (in 30 mL of culture) of *bssA*, *bcrC* and 16S rRNA transcripts were obtained by calculating the amount of copies per μL (eq. 2) contained in 150 ng of RNA.

$$\frac{Copies}{\mu L} = \left[Quantity \cdot \left(\frac{1\ mL}{RNA}\right) \cdot \left(\frac{l}{rt_i}\right) \cdot \left(\frac{rt_f}{cDNA}\right) \cdot DF \right] \quad \text{(eq. 2)}$$

Quantity: qPCR copies/ μL; RNA : volume (mL) harvested for RNA extraction; l: elute (μL) after RNA extraction; rt_i: total RNA (μL) used for cDNA synthesis; rt_f: final volume (μL) of the reverse transcription reaction; cDNA: cDNA (μL) used for qPCR reaction; DF: dilution factor used in qPCR reaction.

The calculated *bssA* and *bcrC* copies/μL, were further normalized by using the 16S rRNA gene as the calibrator: the ratio between *bssA* or *bcrC* and the normalizer,16S rRNA, was calculated. Fold changes were calculated by obtaining the ratio between normalized *bssA* or *bcrC* values of toluene-grown cells to normalized *bssA* or *bcrC* values of benzoate-grown cells (reference cultures) which are subjected to the same oxygen conditions and nitrate concentrations (anaerobic cultures) e.g. AN_T2.5/AN_B2.5, AN_T5/AN_B5, etc (Table S3).

Table S3: Culture pairs used to assess *bssA* and *bcrC* expression from toluene-grown cells relative to *bssA* and *bcrC* expression from benzoate-grown cells.

	Anaerobic conditions	
Experiment	Toluene-grown cells	Benzoate-grown cells
1	AN_T2.5	AN_B2.5
2	AN_T5	AN_B5
3	AN_T10	AN_B10
	Aerobic conditions	
	Toluene-grown cells	Benzoate-grown cells
4	AB_T	AB_B
5	AB_TN	AB_B

2.7. *Peptide detection by LC-MS*

Purified peptides were dissolved in 20 μL of acetonitrile (100%), incubated for 10 min at room temperature and consecutively in an ultrasonic bath 3 x 10 s. Further, the samples were centrifuged and the supernatants were transferred into 12 x 3 mm LC-MS glass vials (Waters Corporation, Milford, CT, USA).

Peptides were then injected into a Nano-HPLC and trapped in a C18-reverse phase column (Acclaim PepMap® 100, 75 μm x 2 cm, particle size 3 μM, nanoViper, Thermo Fisher Scientific, Waltham, MA, USA) for 5 min. Peptide separation by a two-step gradient consisted in 90 min from 4% to 30% of B (B: 80%acetonitrile, 0.1 % formic acid in MS-grade water) and then 30 min from 30% to 55% of B. The temperature of the separation column was set to 35 °C and the flow rate was 0.3 μL /min.

The eluted peptides were later ionized and measured. The MS was set to a full MS/dd-MS^2 mode scan with positive polarity. The full MS scan was adjusted to a MS resolution of 120,000, MS automatic gain control (AGC) target of $3{\cdot}10^6$ ions, maximum injection time for MS of 80 ms and a scan range of 350 to 1550 m/z. The dd-MS^2 scan was set to a resolution of 15,000, AGC target of $2{\cdot}10^5$ ions, a maximum injection time for 120 ms, TopN 20, isolation window of 1.6 m/z, scan range of 200 to 2000 m/z and dynamic exclusion of 30 s.

2.8. *Proteome data analysis*

Proteomic quantification was done using the Top-3 peptide area for approach followed by log2 transformation. Transformed values were normalized manually by dividing the transformed values by the median of each sample. The ratio was further multiplied by the mean of the entire proteome data set (aerobic conditions separately from anaerobic conditions). Fold changes were calculated relative to benzoate-grown cells as described in section 2.6 (Table S3). Down-regulated proteins (negative values) indicate that the protein is more abundant in benzoate-grown cells than in toluene-grown cells. Contrary, up-regulated proteins indicate that the protein is more abundant in toluene-grown cells rather than in benzoate-grown cells. Since fold change of 2 was defined as the threshold, up-regulated proteins were defined by $n \geq +1$ and down-regulated proteins were defined by $n \leq -1$. Student´s T- test was the statistical method chosen to calculate significance between data. All T-test values below 0.05 were considered as statistically significant.

© 2020 by the authors. Submitted for possible open access publication under the terms and conditions of the Creative Commons Attribution (CC BY) license (http://creativecommons.org/licenses/by/4.0/).
